# A large-scale survey of the postmortem human microbiome, and its potential to provide insight into the living health condition

**DOI:** 10.1038/s41598-018-23989-w

**Published:** 2018-04-10

**Authors:** Jennifer L. Pechal, Carl J. Schmidt, Heather R. Jordan, M. Eric Benbow

**Affiliations:** 1 Michigan State University, Department of Entomology, East Lansing, MI 48824 USA; 2 Wayne County Medical Examiner’s Office, Detroit, MI 48207 USA; 3 University of Michigan, Department of Pathology, Ann Arbor, MI 48109 USA; 4 Mississippi State University, Department of Biological Sciences, Mississippi State, MS 39762 USA; 5 Michigan State University, Department of Osteopathic Medical Specialties, East Lansing, MI 48824 USA; 6 Michigan State University, Ecology, Evolutionary Biology, and Behavior Program, East Lansing, MI 48824 USA

## Abstract

The microbiome plays many roles in human health, often through the exclusive lens of clinical interest. The inevitable end point for all living hosts, death, has its own altered microbiome configurations. However, little is understood about the ecology and changes of microbial communities after death, or their potential utility for understanding the health condition of the recently living. Here we reveal distinct postmortem microbiomes of human hosts from a large-scale survey of death cases representing a predominantly urban population, and demonstrated these microbiomes reflected antemortem health conditions within 24–48 hours of death. Our results characterized microbial community structure and predicted function from 188 cases representing a cross-section of an industrial-urban population. We found strong niche differentiation of anatomic habitat and microbial community turnover based on topographical distribution. Microbial community stability was documented up to two days after death. Additionally, we observed a positive relationship between cell motility and time since host death. Interestingly, we discovered evidence that microbial biodiversity is a predictor of antemortem host health condition (e.g., heart disease). These findings improve the understanding of postmortem host microbiota dynamics, and provide a robust dataset to test the postmortem microbiome as a tool for assessing health conditions in living populations.

## Introduction

The human body is a host for a network of microorganisms in constant flux for each of the estimated 7.5 billion people on Earth. The composition and role of the human microbiome has been extensively studied to evaluate human health^1,2^. The microbiota of living hosts is highly influenced by their environment, presence or absence of disease, development, illicit or prescribed chemical substances, and nutrition^3–5^. These elements are responsible for microbial community heterogeneity within and across host populations over time^6–8^. Yet, there is a limited understanding of the human microbiome after death. Decomposition is a complex biochemical process dominated by predictable patterns of decay caused by enzymatic reactions, and loss of the processes that help maintain cellular integrity^9^. Previous work has documented a dynamic, stochastic community of microorganisms existing on a now primordial resource (e.g., no host immunity) that undergoes dispersal, local diversification, and competitive interactions after host death^10,11^. These definitions of human microbiota spatial and temporal shifts throughout decomposition, however, have resulted from work conducted at anthropological facilities^10,12,13^. These facilities adhere to stringent legal requirements for donor programs (e.g., no communicable diseases or antibiotic resistant infections), and some anthropological research facilities do not accept autopsied bodies. An additional bias is that those most likely to donate their bodies to medical sciences represent older sociodemographics groups (white, male, >65 years old)^14^. Hence, donor programs have encompassed a limited population. There is a critical need to test the robustness and applicability of previous work by expanding postmortem microbiome research to other demographics of our communities^15^.

In this study, we present a large-scale assessment of the human microbiome after death to determine if postmortem microbial communities abide by the ecological principles established in the living host^1,7,8^. Since microbial communities persist after host death^10,12^, we hypothesized the initial postmortem microbiome would be a reflection of the host microbiome preceding death in a manner that correlates with health status, which could be valuable for comprehensive human microbiome surveillance. Samples were collected in Detroit, MI without targeted groups of clinical interest; thus, microbial communities were surveyed across a variety of death circumstances. Each postmortem interval was estimated based on taphonomic characteristics or corroborated eyewitness reports, with cases ranging from less than 24 h to greater than 73 h postmortem. There was variation in age, sex, health, socioeconomic status, and access to medical care. It is imperative to highlight that previous human microbiome work has intensely surveyed individuals of European origin and middle to upper socioeconomic levels^15^. Our dataset, with populations in the urban, industrial Midwest, provides a larger and more broadly representative source of host communities that are necessary for investigating postmortem microbiomes (Table 1). Our second goal was to provide a cross-sectional assessment of microbial composition in an urban and largely underserved population. Microbial taxonomic profiles from 188 routine death investigation cases were generated from targeted amplicon sequencing (16s ribosomal RNA gene). Predicted functional profiles were also determined with the Phylogenetic Investigation of Communities by Reconstruction of Unobserved States (PICRUSt) pipeline to estimate community gene content based on available sequenced amplicons^16^. The combined composition and predicted functional profiles were modeled in relation to anatomic sampling location, population demographics, and estimated postmortem interval. Our results demonstrate strong niche differentiation of the postmortem microbiota among anatomic habitats, and discrete community turnover patterns that correspond to the estimated time since death from a robust and variable dataset. Moreover, our results suggest antemortem microbial communities persist after death and may provide utility to indicate the state of human health.

**Table 1:** Summary of study aims (including sequencing approach), sample sizes, and case demographics for studies that have characterized the human postmortem microbiome. ARF = Study conducted at an anthropological research facility.

Reference	Study Aim	Sample size (Total # Subjects)	Median Age (Range)	Ethnicity	Sex Ratio (Male: Female)	Manner or Cause of Death	Estimated PMI or Decomposition Time	Geographic Location of Study
Current Dataset	Large-scale survey of the microbial community structure (16S rRNA amplicon; MiSeq) and predictive function (PICRUSt) from ears, eyes, nose, mouth, rectum and umbilicus during routine death investigation.	188	43 years (18–88 years)	90 Black, 98 White	1.3	Accident, Homicide, Natural, Suicide	1–73+ h PMI	Michigan
Hyde *et al*.^12^	Assess changes in the bacterial community (16S rRNA amplicon; 454 Pyrosequencing) of the gut at pre-bloat and end of bloat.	2	52 & 68 years	2 White	2.0	Natural, Carbon monoxide poisoning	*ca*. 30 days decomposition	Texas (*ARF*)
Can *et al*.^42^	Compare extraction methods for microbial communities (16S rRNA amplicon; 454 Pyrosequencing) of the spleen, liver, brain, heart and blood.	11	47 years (20–67 years)	No data provided.	2.7	No data provided.	20–240 h PMI	Alabama
Hauther *et al*.^25^	Targeted qPCR of three gut bacteria (*Bacterioides*, *Bifidobacterium*, and *Lactobacillus*) from the proximal large intestine during decomposition.	12	65 years (51–88 years)	12 White	0.5	Natural	9–20 days decomposition	Tennessee (*ARF*)
Damann *et al*.^39^	Succession of postmortem bacterial communities (16S rRNA amplicon, 454 Pyrosequencing) from bones (lower rib).	12	57 years (26–88 years)	No data provided.	11.0	No data provided.	571–18,918 accumulated degree days decomposition	Tennessee (*ARF*)
Metcalf *et al*.^10^	Characterization of the postmortem microbial communities (16S rRNA amplicon, 18S rRNA amplicon, ITS, MiSeq, HiSeq) of the skin during decomposition in two seasons.	4	No data provided.	No data provided.	No data provided.	No data provided.	0–82 days decomposition (Spring); Winter data not provided.	Texas (*ARF*)
Johnson *et al*.^13^	Survey of postmortem microbial communities 16S rRNA amplicon; MiSeq) from the nose and ears for machine learning analytical approaches to estimate the postmortem interval.	21	No data provided.	No data provided.	No data provided.	No data provided.	0–800 accumulated degree days decomposition	Tennessee (*ARF*)
Javan *et al*.^43^	Survey of microbial community structure (16S rRNA amplicon, MiSeq) from the blood, brain, buccal cavity, heart, liver, and spleen.	28	48 years (17–82 years)	4 Black, 1 Latina, 23 White	1.2	Accident, Natural, Gunshot (unspecified if homicide or suicide)	3.5–240 h PMI	Alabama & Florida
Javan *et al*.^32^	Survey of postmortem microbial communities 16S rRNA amplicon; MiSeq) from the liver and spleen.	46	41 years (16–82 years)	7 Black, 1 Latina, 37 White	1.6	Accident, Homicide, Natural, Suicide, Undetermined	4–78 h PMI	Alabama & Florida
Debryun *et al*.^24^	Characterization of postmortem microbial communities (16S rRNA amplicon, MiSeq) of the caecum.	4	(62–67 years)	4 White	No data provided.	Natural	0–800 accumulated degree days decomposition	Tennessee (*ARF*)
Adserias Garriga *et al*.^44^	Survey oral postmortem microbial communities (16S rRNA amplicon; MiSeq).	3	27, 80, 81 years	3 White	0.5	No data provided.	0–12 days decomposition.	Tennessee (*ARF*)

## Results

The dataset consisted of samples collected in 2014–2016. Our dataset represents an underserved population from an urban, metropolitan city in the Midwest that is not typically assessed in studies with living subjects (Figs S1, S2 and Table S1). In 2015, 40% of Detroit’s population was at or below the poverty threshold with a median household income of $25,764, which is 54% below the national median^17^. Access to medical care for individuals living in Detroit is more limited than the general population at the state or national level. Approximately 18.9% of the population is a person (under the age of 65 years) without health insurance compared to 6.3% of the population in Michigan and 10.2% of the national population^17^. Further, logistical and financial constraints, such as affordable and reliable transportation, prevent those individuals in the Detroit Metropolitan area with health insurance from seeking medical attention in times of need.

Accidental and natural deaths comprised a majority of the sampled cases at 38% and 30%, respectively, while homicides (20%) and suicides (12%) accounted for the remaining deaths. The average ( ± SD) age of the cases at the time of death in this dataset was 44 ± 15 years. Deaths from homicide had a lower average age (35 ± 13 years), while natural deaths had a higher average age (53 ± 11 years). Accidents and suicides were 40 ± 14 years and 49 ± 17 years, respectively. The majority of cases in this dataset (87%) were classified with a postmortem interval of less than 48 hours after host death (Table S2). Cases with an increased postmortem interval (e.g., greater than 49 hours) were primarily male (64–82%; Table S2).

**Figure 1:**
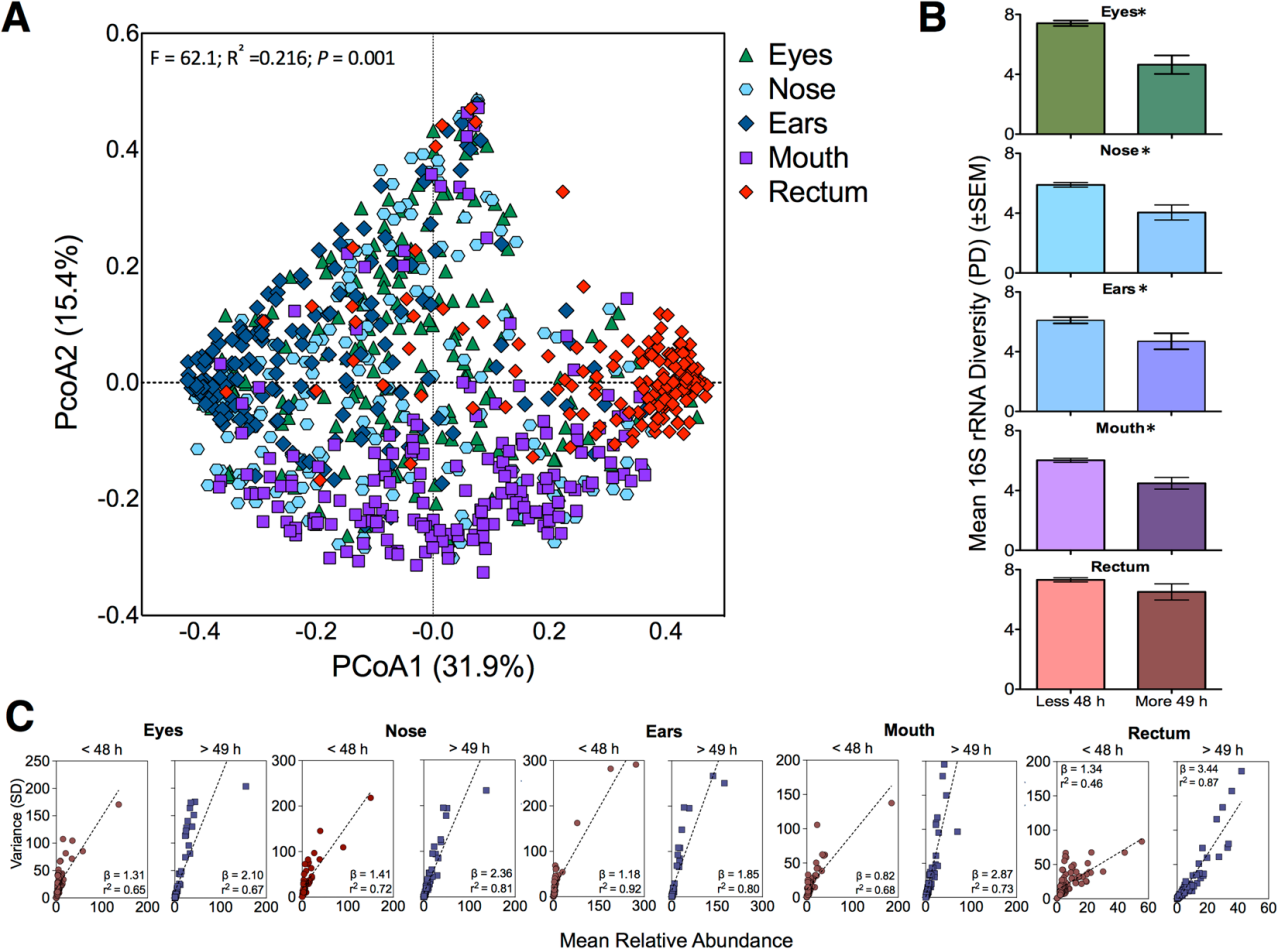
Host filtering of the postmortem microbiome. (**A**) The principal coordinate analysis (PCoA) indicates differences among anatomic location microbiota. PERMANOVA detected significant differences (P < 0.05) among anatomic locations, and all pairwise differences were statistically significant with p-value adjusted for FDR (P < 0.001). (**B**) Faith’s phylogenetic distance (PD) (mean ± standard error mean) was statistically reduced (Mann-U t-test, P < 0.05) for each anatomic location, except the rectum, after 48 h postmortem. (**C**) The relationship between the mean relative abundance and variance (SD) for taxa that were >0.25% relative abundance demonstrated decreased variability in the microbiota in the first two days after death.

We performed a permutational multivariate analysis of variance (PERMANOVA) to identify case covariates structuring the microbial communities based on weighted UniFrac distances (Table S3). These results demonstrated that anatomic habitat was the predominant factor influencing community composition variation, as has been demonstrated in living hosts^6^. A principal coordinate analysis (PCoA) confirmed the variability in community composition was structured by anatomic location (Fig. 1A; Fig. S3). Further, we did not observe community homogenization across body habitats as decomposition progressed (Fig. S4). This suggests that discrete anatomic locations were not contaminated by postmortem transmigration at this decomposition scale (majority of cases with an estimated PMI of <72 h), and microbial community assembly and dynamics were driven by a selection-dominated regime. Thus, we constrained the remaining analyses within anatomic location.

Initially, each alpha-diversity metric was tested for significant differences in microbial composition against age and BMI (Figs S5 and S6). Bacterial phylogenetic diversity revealed a significant decrease in community richness as decomposition progressed within anatomic location, except within the rectum (Fig. 1B; Mann-U t-test, P < 0.05). This suggests that community succession occurred on a short temporal scale. To further explore whether taxon abundance was a predictor of stability within postmortem interval estimates, we tested taxa mean relative abundance and variance over time (Fig. 1C). We observed a linear power-law relationship regardless of anatomic habitat or estimated time since death; however, the slope indicated reduced variation within the first two days since death. This suggests increased microbial community stability in the first two days after death compared to later communities as determined by estimated time since death (>49 h)^18^, which is congruent with the PCoA results. Despite the reduced microbiome configuration (e.g., fewer taxa) during longer estimated postmortem intervals (>49 h), inter-specific competition or host filtering may explain the increased variance of the remaining taxa two days after death.

**Figure 2:**
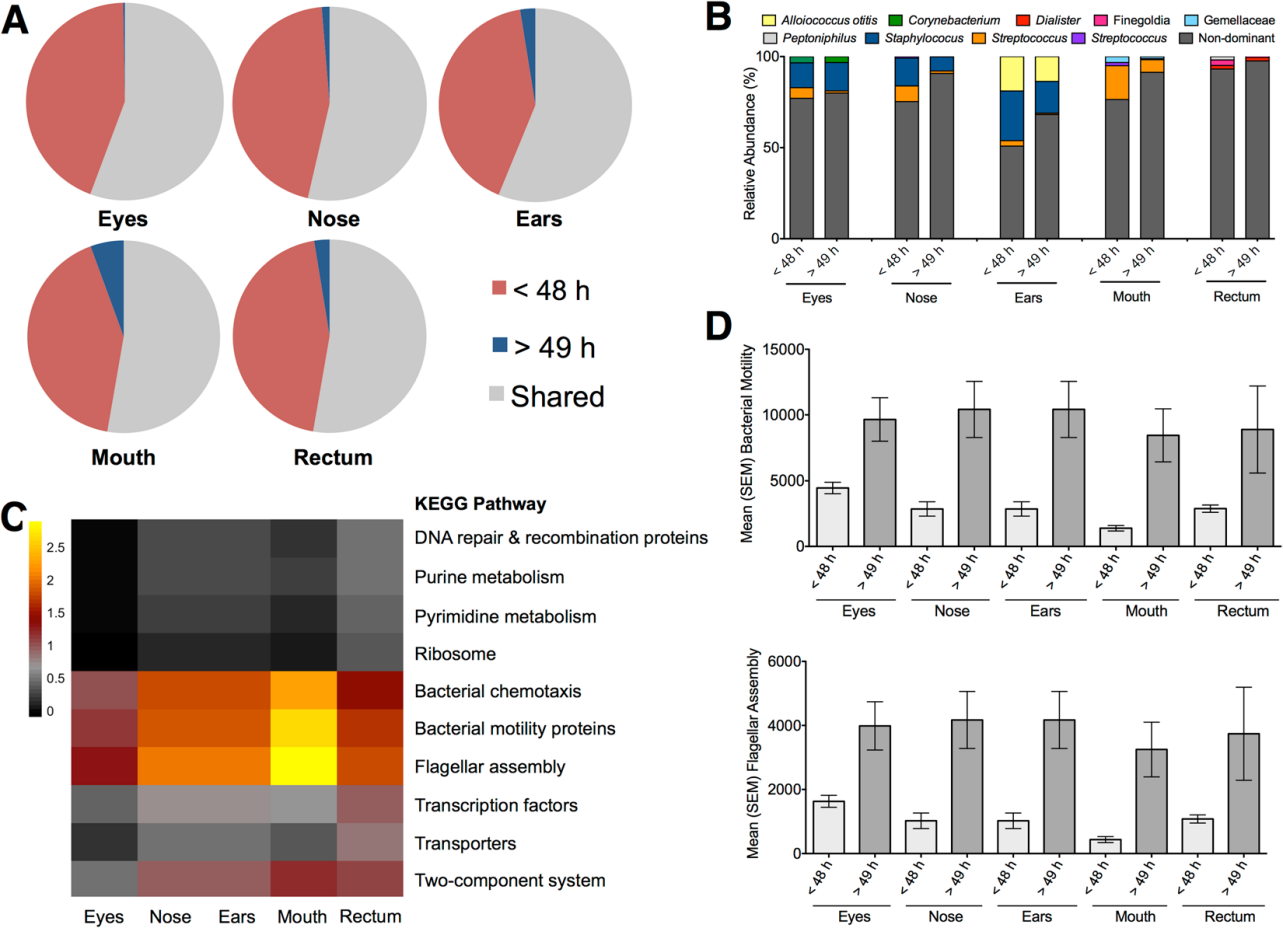
Microbial community profiles from death investigation. (**A**) The proportion of shared OTUs demonstrated substantial taxon overlap among anatomic areas throughout decomposition (>53%), and decreased unique taxa 48 h after host death. (**B**) The relative abundance of predominant taxa changed within two days after death. (**C**) These changes were also detected in the log_2_-transformed fold changes of indicator KO pathways based on *in silico* functional pathways. (**D**) Bacterial motility and flagellar assembly had the greatest increase in relative abundance (mean ± standard error mean) over time by 54–84% and 59–87%, respectively, and were statistically different (Mann-U t-test, P < 0.05) between estimated postmortem intervals.

The log_2_ fold change on median adjusted OTUs revealed that successional processes were detectable at the phyletic level (Fig. S7). Actinobacteria and Bacteroidetes decreased during decomposition, while Proteobacteria increased. Additionally, the taxonomic overlap between postmortem interval estimate times represented less than 10% of the OTUs (Fig. 2A). To determine whether specific taxa were driving these successional changes, we explored the relative abundance of predominant genera within anatomic location (Fig. 2B). Taxa commonly detected in living hosts, such as *Staphylococcus* and *Streptococcus*, were identified. For example, *Alloiococcus otitis* was a predominant species identified in the ear, which is a pathogen commonly associated with otitis media^19^. The relative abundance of these predominant taxa decreased within their respective body habitat after two days postmortem, thus demonstrating that the dynamics of non-dominant taxa are important in later decomposition. ANCOM tests identified 13 OTUs with significantly different abundance within each anatomic area at each decomposition scale (as described in detail in the methods). Out of the 13 OTUs identified as significant (Table S4), only three had a median abundance above zero and were prevalent in >30% of all samples: *Streptococcus* (*W* = 1013) was detected in the eyes with higher abundances early in PMI ranges (<24 h, 25–48 h), while *Haemophilus parainfluenzae* (*W* = 734) and *Streptococcus* (*W* = 759) were more abundant in the mouth <24 h and 25–48 h after death.

To test whether predicted functional community profiles shifted during decomposition, we explored the inferred KEGG ortholog (KO) pathways and identified significant functional signatures that varied with time since death. We developed machine-learning models, based on stochastic gradient boosting regressions of KO features^20^ to predict the postmortem interval less than or greater than two days. Model performance within anatomic area, except the rectum, was good based on both model accuracy and areas under the curves in the receiver operating characteristic (ROC) plots being near 1 (Table S5). We observed an increase in cell motility pathways, including chemotaxis, motility proteins, and flagellar assembly, as the postmortem interval increased (Fig. 2C and D), most notably in the mouth.

We also explored relationships between microbial taxa and predicted function to identify key correlations contributing to temporal changes in the community ecology of the microbiomes (Fig. S8). We detected increased functional redundancy in the first 48 h postmortem among anatomic locations (Fig. S8), which suggests that despite the higher taxon diversity in the first 48 h, compared to after 49 h postmortem, there are similar functions being performed across a number of microbial taxa. Since cell motility increased with the postmortem interval, we sought to identify those OTUs with strong positive correlations to either bacterial motility proteins or flagellar assembly (ρ > 0.5). Among these taxa, Enterobacteriaceae were the most frequently correlated OTUs to cell motility.

**Figure 3:**
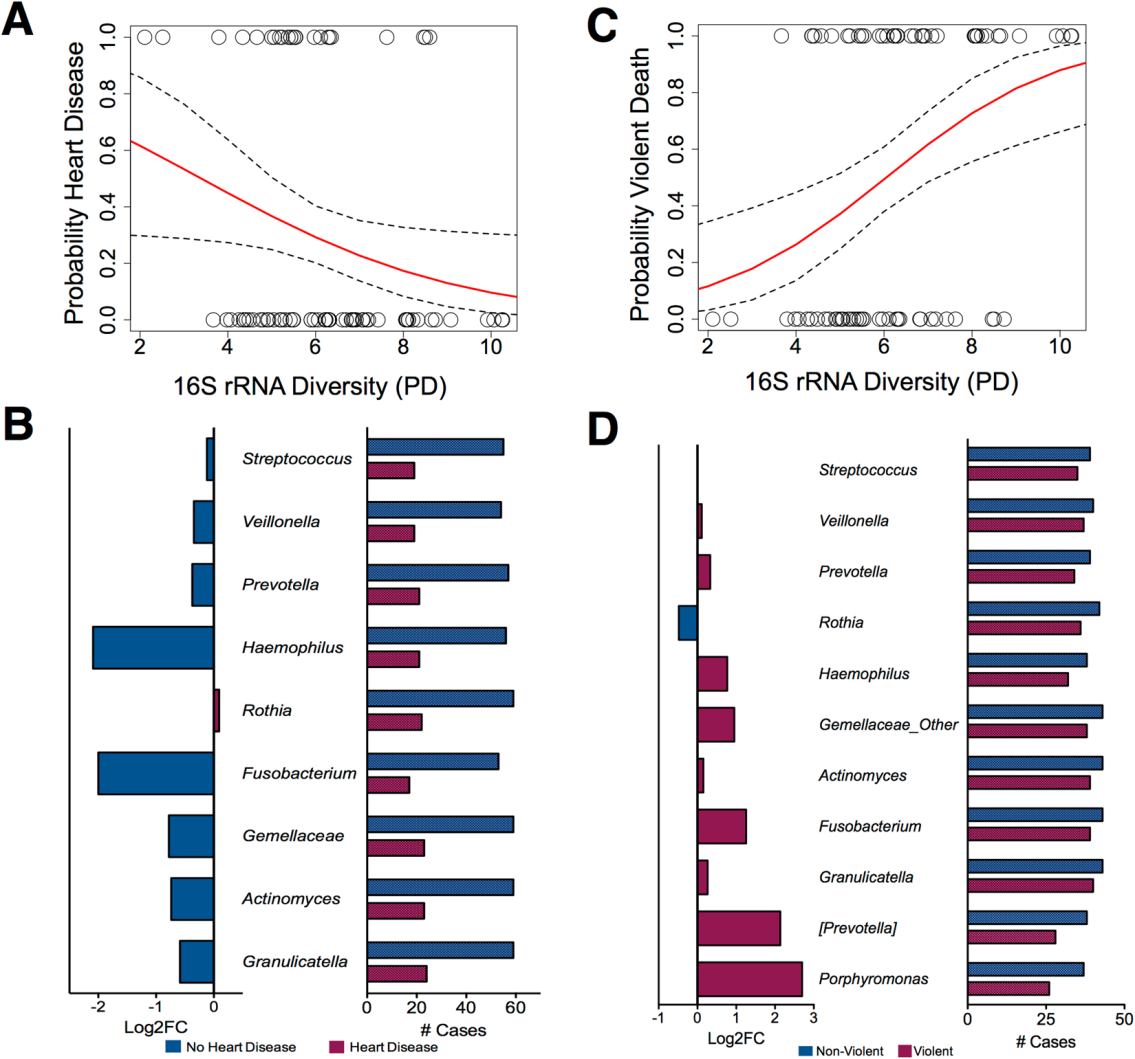
Potential utility of the postmortem microbiome for detecting antemortem health. (**A**) A binomial logistic regression model determined the relationship between Faith’s phylogenetic distance and evidence of heart disease; the odds of a case with a heart condition was 28.8% less likely to occur for each unit increase in phylogenetic diversity. (**B**) The log_2_-transformed fold changes (median) of potential biomarkers for heart disease determined *Rothia* had the only detectable increase in abundance. (**C**) Additionally, the odds of a case resulting from a violent death was 65.2% more likely to occur for each unit increase in Faith’s phylogenetic diversity using a binomial logistic regression model. (**D**) The abundance of *Rothia* was the only taxon detected to increase in non-violent deaths based on log_2_-transformed fold changes (median).

To evaluate whether there were statistical associations between the postmortem microbiome and antemortem health status (Table S6), we performed binomial logistic regression models (with a logit link) to contrast community diversity with the leading cause of death in the US – heart disease^21^. For this example of potential utilization of the postmortem microbiome as a tool for public health, we selected communities less than 24 h postmortem from the mouth, since this anatomic area was the best model for predicting heart disease when compared to models developed from microbial communities of the mouth less than 48 h postmortem (Table S7). Decreased phylogenetic diversity was observed as a significant predictor (P = 0.038) of heart disease (Fig. 3A). The predominant taxa detected during this 24 h window included (Fig. 3B): colonizers, such as *Streptococcus*, *Haemophilus*, and *Veillonella*; anaerobic genera (*Prevotella*, *Fusobacterium*); and *Rothia*, which is a genus commonly found in dental plaque. These taxa are also prevalent in the living host mouth environment^22^. Notably, *Rothia* exhibited the only low, but detectable, increase in abundance for cases with heart disease. This pathogen has been significantly associated with infectious endocarditis^23^. Additionally, individuals whose death resulted from violent circumstances had an increased microbial diversity, as determined by Faith’s phylogenetic diversity (Fig. 3C). These data suggest increased microbial biodiversity may be an indicator of individuals without chronic health conditions, such as heart disease, and could be considered a “healthy cohort” of our sampled population. *Rothia*, again, was the only taxon to be detected in non-violent death cases with a 0.48-fold increase compared to cases resulting from a violent death (Fig. 3D). Thus, *Rothia* in combination with additional community metrics (e.g., diversity) may be an indicator to further explore as a biomarker of hosts with chronic dysbiosis.

Finally, the cross-sectional design provided a unique opportunity to perform a power analysis for the postmortem microbiome. To put the number of cases from our dataset (n = 188) into context, previous longitudinal studies of the human postmortem microbiome have provided data from 2–46 cases^10,12,13,24,25^. We computed the power of detecting a community shift for two groups of unequal sample size for each anatomic location at a significance level of 5%. Our results indicated samples from the mouth had the greatest discriminating power (Cohen’s d = 1.13; power = 1; Table S8). Power analyses showed that a large-scale study, such as ours, is necessary for characterizing postmortem microbiome changes.

## Discussion

Our findings characterize the spatial and temporal heterogeneity of the human postmortem microbiome across a large-scale study. By focusing on the microbiota after death, we highlight that ecological selection is the driving force of community assembly, and the potential to further expand the knowledge of human microbiomes across populations through utilization of an often-overlooked yet ubiquitously available resource – the postmortem microbiome. Analysis of the impact of host and environmental factors from diverse populations will likely provide insights that can promote testing postmortem microbial communities as indicators of human health and forensics. The evaluation of postmortem microbial consortia may transform our understanding of human health among large populations by supporting evidence that long-term surveillance of microbiomes at or within a day of death could have broad public health and forensic relevance.

Further, there are challenges for researchers studying microbial assemblages in complex and uncontrolled environmental conditions. One initial challenge that our group overcame was consistent and reliable sampling during routine death investigation processes. We did so by developing standardized protocols and training investigators and autopsy staff to systematically and consistently collect samples as part of their routine daily caseload. Establishing these methodologies is vital to expand the potential utility of postmortem microbial communities as a surveillance tool for human health or for use in forensics. Another is the potential for cross-contamination to occur during autopsy in the larger offices were multiple autopsies are often conducted simultaneously. However, our results demonstrate there are distinct community differences and succession patterns among all cases. If cross-contamination was of significant biological importance we would expect that microbial communities among cases would be more similar regardless of anatomic area, age, sex, ethnicity, weight status, estimated PMI, antemortem health conditions, or manner of death: we did not find evidence of cross-contamination.

### Cross-Sectional Successional Patterns

Our results demonstrate that despite the fluidic process of decomposition and decay, the body maintains strong niche differentiation among anatomic locations. The umbilicus sample did not provide useful results for the purposes for this study, as indicated by analysis of the beta diversity (Table S2; Fig. S3), and the umbilicus only resulted in samples with adequate post-filtering sequences in half of all cases (50%). Further, we did not conclude there was a homogenization of microbial communities across all anatomic sites as the time since death increased (Figs S4 and S8), as others have noted distinct microbial communties based on anatomic location in living hosts^1,7,26^. However, we acknowledge the skewed sampling to cases with an estimated PMI of less than two days (87%). This is because, as a practical matter, most deaths are reported or discovered a short time after they happen regardless of the size of the community, the vast majority within 48 hours of death. Thus, our dataset is important for establishing a foundational baseline of postmortem microbial characterization that may be useful in future forensic applications because, as far as we know, mid-sized and larger death investigation systems in the United States have comparable practices so that our results may be applicable elsewhere. It is probable that the microbial communities of all anatomic locations become more similar to each other over prolonged periods of decomposition (e.g., weeks to months), or the body becomes more similar to the environmental microbial communities, such as soil or aquatic habitats, as found in other studies^10–12,24,25,27,28^.

The demographics of our population may be influencing the structure and function of the reported microbiome microbial communities. Our dataset is comprised of 52.1% of cases being white only, which reflects the 2016 US Census^17^, that determined the population of Wayne County (MI) is 54.6% (white only). It is important to note that the demographics of this dataset are reflective of the composition of the living population in Wayne County, and not a skewed subset of an *a priori* targeted or self-selecting demographic as is common of many living microbiome studies^1,2,4,8,26,29–31^. The goal of this initially large-scale survey was to identify the covariates most important in structuring microbial communities after death for a population not preselected based on health condition.

The relationship of the alpha diversity metrics with the age indicated a weak negative relationship with diversity metrics for all anatomic locations, except the ears. These results provide initial evidence that there is reduced richness, diversity and evenness in the postmortem microbiome with aging. The same relationships were tested with BMI instead of age, and a weak positive relationship emerged between BMI and the alpha diversity metrics across all anatomic locations. Previous surveys of the postmortem microbial communities for internal organs have also demonstrated sex was nearly significant (p = 0.05) in structuring microbial richness and not organ type, age, estimated PMI, or ambient temperature; however, organ type and sex had significant differences based on Shannon diversity^32^. Results from this dataset (Table S3, Figs S3, S5 and S6) demonstrated manner of death, sex, estimated postmortem interval, death event location, ethnicity, season of death, and body weight (in descending order of significance) were not the most important covariates structuring the microbial communities. It has been argued that ethnicity is a reflection of social and political groupings with little biological basis^33^. Microbial taxon differences have been observed in living individuals with differing self-reported results of race/ethnicity^1,34^, despite ethnicity not being a strong covariate (p > 0.6) structuring our dataset. The weight of an individual has been shown to be important in structuring microbial communities in living individuals^1,5,35,36^. However, the postmortem microbial communities could also be influenced by other factors, such as the built, urban environment, undisclosed health conditions, medications or other environmental chemicals, which were not explicitly tested using this dataset. Future in-depth analysis of the influence of specifics covariates on postmortem microbial communities is warranted.

To test the variability of taxa over decomposition time, we plotted the mean taxon abundance by their variability and determined within each anatomic area the relationships followed Taylor’s (power) law. Mathematically, Taylor’s law should have a slope of two^37^. But it is common within naturally occurring ecological systems to have a slope less than two^38^. A slope less than two indicates that species dominating populations should be less variable in space or time; this relationship of predominate species maintaining their population size could be caused by negative species interactions (direct or apparent competition) or interspecific competition^38^. For our dataset, the combination of increased slopes after two days since death with lower phylogenetic diversity after two days postmortem suggests that negative species interactions could be a potential mechanism of succession.

Further, we documented that most variability over decomposition occurred in the microbial communities of the mouth, while community membership and structure were most consistent in the rectum. These results suggest that the external communities are more subjected to physics and the environmental interactions while internal communities are more influenced by biochemical processes. Despite the dimensionality of our dataset, which may be explained by biotic interactions, stochastic effects, host genetics, or other unknown factors, the prevalence of common taxa and composition detected in the first two days postmortem suggests that microbial communities are not undergoing rapid turnover within 24–48 h of host death. Therefore, we postulate the taxa of the first 24–48 h after death will most represent antemortem microbial communities.

### Comparisons to Previous Postmortem Human Microbiome Studies

Our large-scale survey of the human postmortem microbiome clearly demonstrates that microbial community composition changed among anatomic areas and over time. Notably, our study has approximately three times the number of cases than previous studies, and we report predictive *in silico* function profiles from a large-scale human postmortem database. Due to the large number of cases analyzed (188 cases), we were able to examine the observed power (Table S8), which has not been previously reported in other human postmortem microbiome studies^12,13,24,25,32,39–44^. Differences in the microbial communities less than two days compared to greater than two days after death gave us the most power (>0.8) to detect large effect sizes (d > 0.5) in the mouth and ears. The remaining anatomic locations (eyes, nose, rectum) had less power (<0.8) to detect differences in the microbial communities given the sample sizes (~150 cases < 48 h PMI vs. ~23 cases > 49 PMI). This dataset also has key differences in experimental design, sampling methodologies, and study aims compared to previous studies (see Table 1 for summary). For example, one study profiled bacterial signatures on bones^39^, which is beyond the current scope of this dataset. Other studies focused on surveying the postmortem microbial communities of internal organs and blood^32,42,43^, and thus a direct comparison cannot be made based the anatomic locations sampled for this dataset. We also recognize the time a body has been decomposing (either estimated postmortem interval or time since placement in the field at anthropological facilities) is different when comparing our dataset to previous work from anthropological facility^10,12,13,24,25^ or surveys of the internal organs during death investigation with a smaller sample size (n ≤ 46 cases)^32,43^.

Anatomic location influenced the microbial diversity, with the rectum and eyes having the most phylogenetic diversity while the ears, nose, and mouth having the least diversity (Fig. 1B). These data partially agree with previously reported diversity trends from two bodies that showed increased microbial richness as sampling moved from the upper to the lower the gastrointestinal tract^12^. Another study to survey the microbial communities of the liver, spleen, heart, brain, blood, and mouth of 28 bodies, detected increased richness in the mouth compared to the internal anatomic locations and blood samples^43^. Further, we observed a decrease in alpha diversity metrics (richness and phylogenetic diversity) as the estimated postmortem interval increased (greater than two days after death) in all anatomic locations sampled except the rectum (Fig. 1B). These results are consistent with some previously published data that documented a decrease in microbial diversity in the auditory canal from 21 bodies^13^; only four bodies in that study had repeated sampling events up to 800 accumulated degree days with the remaining 17 bodies having single sampling events to characterize the microbial communities. However, our data do not align with another study that characterized the microbial communities of two cadavers during decomposition in two seasons (spring and winter). Metcalf *et al*.^10^ did not detect statistical differences (alpha = 0.05) in the phylogenetic diversity of skin sites (e.g., hips, biceps, head, groin) over decomposition. Javan *et al*.^32^ also did not detect significant differences (alpha = 0.05) in Chao1 richness or Shannon diversity from samples collected from internal organs (liver, spleen, heart, brain, and blood) and the mouth in cases with estimated PMIs from 3.5–240 h; however, alpha diversity metrics of each sampling location were not presented. Thus, it is undetermined whether the postmortem microbial communities of the mouth were similar in diversity over decomposition time. Overall, the similarity of our dataset to previous studies is strongest when comparing microbial diversity based on topographical distribution. The postmortem microbial communities detected in the mouth were less diverse than those of the rectum. Further, the decline in alpha diversity as decomposition time increased could result from increased competition in the microbial communities later in decomposition (after two days of death) or a successional shift to a more anaerobic taxa configuration.

In each anatomic area, distinct compositional changes were observed at the phyletic level. Actinobacteria and Bacteroidetes abundance decreased after the first two days of decomposition, while Proteobacteria abundance increased after two days of decomposition (Fig. S7A,B). A specific taxon within Bacteroidetes (i.e., *Prevotella)* has been previously detected from mouth microbial communities during the earlier stages of decomposition in both anthropological field studies^12^ and death investigation cases^43^. Additionally, the decline in Bacteroidetes from this dataset is similar to decreased abundance of *Bacterioides* from 12 bodies at an anthropological facility^25^, despite microbial samples being characterized from the proximal large intestine. Hauther *et al*.^25^ performed a targeted study to characterize postmortem dynamics of three common gut taxa during decomposition (9 to 20 days), and also documented a decline in *Lactobacillus* abundance while *Bifidobacterium* did not change during decomposition. We also detected a lower abundance of Firmicutes, specifically *Staphylococcus* and *Streptococcus*, abundance after the first two days of decomposition in all anatomic locations except the nose (Figs 2B and S7A,B). These results are similar to previous studies that documented *Streptococcus* was a predominant taxon in the mouth: during pre-bloat for two bodies decomposing in an anthropological research facility^12^; during the first four days of decomposition for three bodies decomposition in an anthropological research facility^44^; and from 13 death investigation cases with estimated PMI of 10–70 h^43^. ANCOM tests also indicated *Streptococcus* as a potential biomarker in the eyes and mouth during the first two days after death. Additionally, *Staphylococcus* detected in the ear form 21 bodies decomposing at an anthropological facility has been identified as an important indicator for postmortem interval estimates using machine-learning algorithms^13^. However, our results do not align with a previous study that documented an increase in abundance of Firmicutes (*Clostridium* and *Lactobacillus)* from mouth samples. The lack of continuity from the results could stem from the small sample size (two bodies) and the sample collection times, as microbial communities were compared at pre-bloat to end of bloat during decomposition^12^. Nor were our results congruent with another study documenting an increase in Actinobacteria and Firmicutes from the skin microbial communities in later decomposition (four bodies decomposing up to 82 days at an anthropological research facility)^10^. Finally, *H. parainfluenzae* was identified to be a potential bioindicator using ANCOM tests, specifically in the mouth less than 48 h after death. While no study to date has indicated this taxon as a potential bioindicator of postmortem microbial communities, the genus *Haemophilius* is part of the normal bacterial flora of the upper respiratory tract^45^, but can be considered opportunistic pathogens^46–48^, and *H*. *parainfluenzae* has been found in the oral cavities of patients living without periodontal disease^49^. Thus, it is not surprising this taxon commonly found in the mouth of living individuals was an indicator of postmortem intervals less than two days after death.

Constituents of the living host microbial communities, such as *Clostridium* spp., Streptococci, and the Enterobacteria, have been found to be viable up to 48–72 hours after host death^50^. Further, we observed members of Enterobacteriaceae that were OTUs most frequently correlated to cell motility. This result is not surprising given the increased relative abundance of Enterobacteriaceae and other λ-Proteobacteria as decomposition progressed. Predominant taxa within Enterobacteriaceae have swarming behavior, including *Salmonella* and *Proteus*^51^. Thus, it is possible that swarming members of Enterobacteriaceae may be outcompeting other microbial communities at later postmortem intervals. Additionally, the predicted functional profiles suggest the taxa detected two days after death (chemotaxis, motility proteins, and flagellar assembly) may have competitive advantages, such as niche colonization or nutrient acquisition, that result in reduced diversity, yet increased motility as determined by *in silico* functional pathways. Hence, it is not unreasonable to postulate a better understanding of the postmortem microbial communities could provide invaluable insight to human health for the living; assuming that there is not significant change within 24–48 h after death, such as extreme temperature changes that could impact the growth of particular microbial taxa^52^.

### Antemortem and Postmortem Microbiome Linkages

Researchers seeking to better understand the postmortem microbial community dynamics must first determine whether the postmortem microbiota adequately represent microbial populations colonizing the human body prior to death. If this statement holds true, this under-utilized resource could act as a widespread tool for assessing antemortem health, as we have argued in this paper. Previous precedent for use of the postmortem microbiome as a surveillance tool for antemortem health conditions has occurred in tuberculosis research and viral infections^53–55^. We found evidence that cases with heart disease had decreased microbial community diversity (Fig. 3A). This suggests that individuals with heart disease have a reduced microbial configuration, and thus this chronic health condition may be impacting the host microbial biodiversity. As chronic health conditions have been previously reported to impact microbial diversity in living individuals^2,56,57^. The results of a separate binomial logistic regression for violent deaths (e.g., blunt force trauma, gunshot wound, vehicular accident) suggest that cases resulting from violent deaths had increased phylogenetic diversity (Fig. 3C). We recognize that heart disease typically appears later in life (median age = 53 years) and is chronic, while cases from violent deaths tended to be younger individuals (median age = 38 years) (Table S6), and thus may be a correlating factor contributing to the trends observed. Additional studies that follow microbial communities near, at and after death are necessary to more quantitatively link the associations we report here; however, the logistics of such studies will be very challenging.

Based on these results, we are suggesting that utilization of the microbiome after host death could be an important step toward human health surveillance among other practical applications, mainly forensics, biosecurity and precision medicine. While assessing changes in the postmortem microbiome within the first 24 h of death would be ideal, this would require waiting for death and sampling immediately after death, circumstances of substantial ethical concern and logistic difficulties. However, the alternative approach is to incorporate microbiome sampling during death investigation^58^, where samples for microbes are often routinely collected to fulfill forensic microbiological protocols, including but not limited to detection and confirmation of microbes of clinical importance (e.g., bacterial meningitis), or biological agents in suspected biocrimes (e.g., *Bacillus anthracis*). This is the approach that we took for this study, and then identified changes in the microbiome within and after 24 h of death.

To determine the potential of the postmortem microbiome as a health surveillance tool, it is important to first characterize how the microbial communities vary among different postmortem time frames and anatomic areas and identify major changes related to time since death. To assess this potential use, it was important to model the factors and consistency of mechanisms that mediated microbial community dynamics (structure and predictive function) after host death. The detection of potential pathogens and community stability suggests a carryover between living and deceased host microbiota within 24 h of death. Hence, we argue the postmortem microbiota has the promise to identify disease entities that often remain unknown after death, and identifying these biomarker(s) of antemortem health condition would overcome challenges associated with microbiome studies of the living (e.g., limited or targeted sample size and non-invasive sampling). As this and future datasets expand, it is conceivable that resulting data from the postmortem microbiota could provide insights into the health of the community, and even public health intervention if warranted.

Additionally, the data suggest that perhaps individual taxon biomarker(s) will not be as strong of an indicator of health condition when compared to the community of microorganisms associated with the individual (e.g., diversity). However, we detected bacteria in the first 24 h after death that are commonly found in the human microbiota of living individuals^1,59^. This carryover in taxa is important because it indicates a link between ante- and postmortem (<24 h PMI) microbial communities. For example, there were 83 cases with *Streptococccus*: 59 of them had *Streptococccus* in individuals without heart disease, while 24 had *Streptococccus* in cases with heart disease. *Haemophilus* and *Fusobacterium* were two-fold more abundant in healthy individuals where as *Rothia* was 0.09 times more abundant in heart disease cases. Many species of *Rothia* are opportunistic pathogens^23,60^, so it is possible we are detecting infections of individuals that correspond to the subtle abundance increases in unhealthy individuals. Ultimately, we anticipate that the postmortem microbiota could become a valuable resource for informing human health outcomes.

### Potential Societal Impacts from the Postmortem Microbiome

Information from our current dataset could have a direct effect on living individuals by providing a means to broadly survey dysbiotic microbiomes associated with human health status, including chronic conditions, such as obesity, diabetes, and asthma, and helping develop strategies for delivering medical care to diverse or low socioeconomic status populations^1,59^. Here we show that the microbial communities change with time since estimated death, and the consortia within 24 h of death likely represent antemortem health conditions and dysbiosis. This community stability within the first 24–48 h after host death could be of importance for human health and pathogen surveillance and forensics, especially with bacterial species that are difficult to culture with conventional methods. After 48 h the value of postmortem communities for health surveillance may become more limited in this context. Our results indicate the postmortem microbiome could be used as a tool to conduct surveys beneficial to public health, especially in sociocultural areas where there has been consistent failure to understand health states of medically underserved population.

Overall, our study shows that postmortem microbial community dynamics from an urban population reflects a range of human demographics, sociocultural conditions, and life styles. Previous work has provided longitudinal characterizations of the host postmortem microbiota in controlled environments (e.g., anthropological research facility), where body donations are *a priori* selected based on stringent criteria. These studies often encompass a limited population (up to 50 cases), thus due to the size of our large-scale dataset it allows a robust characterization of the microbial communities after death, and provide data from wide swath of the population not often studied in human microbiome work. Therefore, our dataset contributes empirical evidence that advances the understanding of the postmortem microbiota and their function in a variable population. Further, we postulate the postmortem microbiome, within 24–48 h of death, is a reflection of the host microbiome preceding death. We identified a postmortem microbiota metric as a significant predictor of an important antemortem health status (and the leading cause of death in the US) – heart disease. Thus, this novel approach may provide a comprehensive tool with utility to indicate the state of human health in a way that can be sampled during clinical investigation in a range of deaths, from chronic and natural to sudden and violent.

## Materials and Methods

We collected microbial community samples from human cases during routine death investigation in a major, metropolitan US city located in the industrial Midwest (Wayne County, Michigan). Our non-targeted, cross-sectional design allowed us to explore postmortem microbial dynamics in an underserved community of our global population, and investigate the potential of postmortem microbial communities as a surrogate for the living host health condition.

### Data Availability

The data reported in this paper can be found summarized in the online-only methods section. Sequence data to support the findings of this study were archived through the European Bioinformatics Institute European Nucleotide Archive (www.ebi.ac.uk/ena) under accession number: PRJEB22642. No statistical methods were used to predetermine sample size, as the data included here were derived from samples collected during routine death case investigation at a Medical Examiner’s Office, thus the case demographics were outside of our control. No randomization or blinding of experiments was performed, as no treatment or phenotype groups were included for this study.

### Case Metadata Information

Microbial samples were collected as part of routine death investigation by the Wayne County Medical Examiner’s Office (MEO) located in Detroit, MI, USA (Fig. S1). We obtained the following case information (Fig. S2; Table S1): sampling date (season); sex; ethnicity; estimated age (years); location of death (indoors, outdoors, hospital, vehicular); and anatomic region (see Sample Collection for additional details). Additionally, from each autopsy report we collected data from each case about the manner of death and estimated postmortem interval (PMI). Each manner of death and PMI estimate was determined by a board certified forensic pathologist at the time of autopsy; PMI estimates were determined with taphonomic markers (e.g., rigor mortis, livor mortis) and/or corroborated eyewitness reports. The body mass index (BMI; kg/m^2^) was calculated for each decedent and classified into obesity classifications as established by the World Health Organization (Fig. S2): <18.5 = underweight; 18.5–24.9 = normal weight; 25.0–29.9 = overweight; 30.0–34.9 = class I obesity; 35.0–39.9 = class II obesity; ≥40.0 = class III obesity. Finally, the antemortem health status of each case was determined from results of the autopsy or known medical history. We categorized the presence of antemortem health conditions into the following classifications: heart disease or if the decent died in a violent manner (e.g., sharp force trauma, gunshot wound or vehicular crash). Criteria for being included in this dataset were as follows. The decedent must be an adult (18+ years old); male or female; black or white; and have a known manner of death (all indeterminate cases were excluded from this dataset).

### Sample Collection

Trained personnel at the Wayne County MEO collected microbial samples aseptically using DNA-Free sterile cotton-tipped applicators (25–806 1WC FDNA, Puritan^®^, Guilford, MA, USA). Individual microbial communities were sampled from six external anatomic locations for each case (Fig. S2): the external auditory canal, eyes, nose, mouth, umbilicus, and rectum. For each anatomic location, an individual swab was physically rubbed while rotating the swab for 3–5 seconds in order to thoroughly sample the associated microbial community. The cotton end of the applicator was immediately placed in an individual sterile microcentrifuge tube (1.7 ml, 87003-294, WVR^®^, Radnor, PA, USA) filled with 200 μl of molecular grade ethanol (100%, BP2818-4, Fisher Scientific, Waltham, MA, USA). Samples were stored at −20 °C until further processing.

These areas were chosen based on practicality and accessibility during investigation. For instance, to sample the ears, eyes, nares, and mouth, the host clothing does not need to be removed or is easily moved for swabbing. Furthermore, the microbial communities in these areas are more likely to represent a “microbial clock” compared to internal communities (*e*.*g*., gastrointestinal tract) that are influenced by diet or weight status^5,29^.

Institutional Review Board (IRB) review is not required for research on human remains from deceased individuals. For IRB purposes, they are considered under the same rules as those applied to autopsy specimens, and are not considered human subjects research. There was no intentional tissue sampling or removal of tissue at the time of microbial collections. Microbiological sampling is an established procedure in pathologic diagnosis.

### DNA Isolation from Swab Samples

Genomic DNA (including microbial) was extracted, under aseptic conditions in a biological safety cabinet, from the applicator tips in individual reactions using the PureLink^®^ Genomic DNA Mini Kit (Thermo Fisher Scientific, Waltham, MA, USA) following the manufacturer’s instructions with the following modification: 15 mg/mL of lysozyme was added during the lysis step for reaction^41^. DNA was quantified using the Quant-iT dsDNA HS Assay kit and a Qubit 2.0 (Thermo Fisher Scientific, Waltham, MA, USA). The DNA elutions were stored at −20 °C until submission for high-throughput sequencing (HTS); remaining samples after submission for HTS were stored at −80 °C.

### 16S rRNA Gene Amplicon High-Throughput Sequencing and Processing

All microbial DNA was sequenced using Illumina MiSeq of 2 × 250 bp paired-end reads at the Michigan State University Genomics Core Facility (East Lansing, MI, USA). 16S rRNA gene amplicon library construction and sequencing was performed using a modified version of the protocol adapted for the Illumina HiSeq2000 and MiSeq; V4 regions of the 16S rRNA gene amplicon region were amplified with region-specific primers that include Illumina flowcell adapter sequences [515 f (5′ GTGCCAGCMGCCGCGGTAA) and 806r (5′ GGACTACHVGGGTWTCTAAT)]^61^. PCR products were batch normalized using Invitrogen SequalPrep DNA Normalization Plates and recovered amplicon products pooled. The library pool was quality controlled and quantified using a combination of Qubit dsDNA HS, Caliper LabChipGX HS DNA, and Kapa Illumina Library Quantification qPCR assays. After cluster formation on the MiSeq instrument, amplicons were sequenced with custom primers complementing amplification primers to avoid primer sequencing. The pool was loaded on an Illumina MiSeq standard flow cell (v2) and sequenced in a 2 × 250 bp paired end format using a MiSeq v2 500 cycle reagent cartridge. Custom sequencing and index primers described in Kozich *et al*.^62^ were added to the appropriate wells of the reagent cartridge. Furthermore, the filtering parameters for sequence classification was optimized using settings recommended by Caporaso *et al*.^63^ as phylogenetic diversity among Operational Taxonomic Units (OTUs) with low abundance can be affected by the filtering parameters^64^. Base calling was done by Illumina Real Time Analysis (RTA) v1.18.54 and output of RTA was demultiplexed and converted to FastQ format with Illumina Bcl2fastq v1.8.4.

#### Quantitative Insights Into Microbial Ecology (QIIME)

Raw fastq files were assembled, quality-filtered, and analyzed using the default settings [join_paired_ends.py (-m SeqPrep); split_libraries_fastq.py (-q 19)] in QIIME (version 1.8.0 for Macintosh), as described in our previous work^41,65^ and others^66^. After quality control, the remaining high-quality sequences were binned into OTUs at a 97% sequence similarity cutoff using UCLUST^67^. Assembled sequence reads were classified into OTUs on the basis of sequence similarity; the highest-quality sequences from each OTU cluster were taxonomically assigned using the RDP classifier after chimera identification and removal via ChimeraSlayer^68,69^, and identified using BLAST against reference sequences from the May 2013 release of the Greengenes 97% reference dataset http://greengenes.secondgenome.com/^70–72^ using a closed reference strategy (pick_closed_reference_otus.py). Representative sequences of all OTUs were aligned to the Greengenes reference alignment using PyNAST^73^. Singleton OTUs and any remaining low abundance OTU’s making up <0.0005% of reads in the total dataset were removed, as recommended for Illumina generated data^10,74^. Also, plant Chloroplast and mitochondria sequences were filtered from the dataset using the filter_taxa_from_otu_table.py script. Samples were rarefied to 1,000 sequences to remove sample size bias on community composition; microbial libraries containing fewer than 1,000 sequences were removed, as previously performed by our group and others^10,41^.

#### Phylogenetic Investigation of Communities by Reconstruction of Unobserved States (PICRUSt)

A computational approach was used to make predictions regarding the functional composition of the microbiome based on the 16S rRNA amplicon sequences^16^. OTU abundances were normalized to known or predicted 16S rRNA copy number abundances against Greengenes 13.5 database (May 2013 release). Using KEGG pathway metadata, KEGG orthologs (KO) were predicted and collapsed into hierarchical KEGG pathways (level 3) by their function^75^. All PICRUSt analyses were performed using the online Galaxy version (https://huttenhower.sph.harvard.edu/galaxy/) using default settings.

### Statistical Analyses

#### 16S rRNA Community Structure Analyses

We characterized community-level microbiome composition variability among cases to explore individual spatial and temporal heterogeneity of the postmortem microbial communities. Initially, we included the umbilicus samples in this dataset, but beta diversity patterns, evaluated using principal coordinate analysis (PCoA) with weighted UniFrac distances that were calculated in QIIME, suggested that the umbilicus did not have a strong unified grouping but rather was related in composition to all non-rectum anatomic locations (Fig. S3). These results were confirmed with a permutational multivariate analysis of variance (PERMANOVA) test that resulted in increased *pseudo-F* and *R*^2^ values when the umbilicus was removed (Table S2); these analyses were performed in R using the *vegan* library^76^ with 999 permutations. The weighted UniFrac metric was used for each PERMANOVA since it takes into account phylogenetic distances among microbes and their relative abundance^77^. Thus, we removed the umbilicus samples from the dataset for the remaining analyses.

Once the umbilicus samples were removed, we used PCoA plots to assess the significance of sex, ethnicity, event location, weight status (as a surrogate of BMI), season, manner of death, and PMI to account for postmortem microbiome composition variability (Fig. S3). The ordinations confirmed that anatomic location was the best covariate explaining the variability in microbial community composition. Additionally, we used PERMANOVA to test for statistical differences in community structure among the fine scale estimated PMIs of <24 h, 25–48 h, 49–72 h, >73 h, or the broad scale estimated PMIs of <48 h, >49 h, anatomic area, sex, ethnicity, death event location, season, weight status and manner of death. Since a PERMANOVA model with multiple factors was needed to test for differences in microbial community composition, we calculated weighted generalized UniFrac distances in R using the *GUniFrac* library^78^. Based on the results from each full model (Table S2), we confirmed that anatomic location was the most important factor structuring the 16S community profiles regardless of estimated PMI scale (fine PMI scale: <24 h, 25–48 h, 49–72 h, >73 h; broad PMI scale: <48 h, >49 h). Thus, each anatomic area was subset into its own individual dataset for subsequent analyses.

Within each anatomic area, we explored beta diversity dynamics of the microbiome after death (Fig. S4). There were distinct temporal patterns within each anatomic location regardless of the PMI estimate. However, based on PERMANOVA models using weighted UniFrac matrix, the broader definition of estimated time since death (less than or greater than two days after death) resulted in increased *pseudo-F* and *R*^2^ values. Additionally, we tested whether there was a significant interaction effect between anatomic areas and estimated PMI (<48 h, >49 h) with a PCoA and PERMANOVA based on weighted UniFrac distances (Fig. S4).

Alpha-diversity metrics [observed OTUs (richness), Chao1, Shannon-Wiener diversity, Heip’s evenness, and Faith’s phylogenetic diversity] were calculated in QIIME at the OTU level. Nonparametric one-way analysis of variance (Kruskal-Wallis, ANOVA) with multiple comparisons after Dunn’s multiple comparisons or Mann-U t-tests in Prism 5.0 f (OSX, GraphPad Software, Inc., La Jolla, CA, USA) was used to evaluate how diversity, richness and evenness metrics changed after time since host death.

Temporal stability of the postmortem microbiome was assessed using regression analysis. Specifically, we calculated the relationship between taxon mean relative abundance (*m*) and its variance (*V*) after death; both *a* (sampling parameter) and *b* (index of species aggregation) are positive constants. If a scaling relationship (e.g., *V* = *am*^*b*^) was detected, this would imply aggregation in the temporal distribution according to Taylor’s Power Law^37^. We selected taxon for this analysis with >0.25% relative abundance in the population at each PMI estimated range^26^. We then evaluated if there were specific taxa driving the temporal succession patterns within each anatomic area since host death. Shared OTUs found in both samples less than and greater than two days were identified, as were the OTUs that were unique to each estimated PMI (<48 h, >49 h). Proportional representation (% of rarefied sequence reads) of the postmortem microbiome at the phylum level was calculated across anatomic locations (Fig. S7); each column represents an individual case with time since death increasing from left to right. All phyla outside of the top four most abundant (Actinobacteria, Bacteroidetes, Firmicutes, and Proteobacteria) were grouped together as “Rare Phyla”. The log_2_ fold change between estimated PMI (<48 h, >49 h) was calculated on median adjusted OTUs within each of the four most predominant phyla. Further, the proportional representation (% of rarefied sequence reads) of the postmortem microbiome on a generic level was calculated across anatomic locations. The three most abundant genera within each anatomic location were identified and their relative abundance was plotted based on estimated time since host death. The final assessment of temporal stability within anatomic area was determined using density plots for weighted UniFrac distances (Fig. S7). These plots showed the majority of postmortem microbial communities overlapped during decomposition, but the mouth had the most distinct separation between communities when comparing less than two days to those greater than two days since death.

We initially used a machine-learning algorithm to identify important OTUs for classifying the postmortem interval groups. Stochastic gradient boosting was implemented in R using the *caret* library^79,80^, as these algorithms can model complex non-linear relationships. For each model, we created training and validation data sets from given data (80/20 ratio), and used a binary predictor (broad scale PMI estimates). The performance of each model was evaluated based on gradient boosting model (gmb) accuracy, built with 10-fold cross-validation, and an area under the receiver operating characteristic (ROC) curve (Table S3). Then to assess differences from the whole microbial (bacteria and archaea) community of each estimated PMI within anatomic location, we tested for differential abundances of taxa using analysis of composition of microbiomes (ANCOM)^81^ in QIIME2 (2017.12) using the q2-composition plugin^61,82^.

#### Predictive Functional Profile Analyses

Using a similar analytical approach, as we previously described for the microbial community structure analysis, we used stochastic gradient boosting to identify potential important KO pathways for predicting broad scale PMI estimates. Performance for variable selection of each PMI grouping was determined using gbm and ROC metrics (Table S3). In general, the predicted functional profiles had better model performance and thus we determined linear discriminant analysis effect size (LEFSe) using the default settings in the online Galaxy version to evaluate consistency in feature selection. Once we identified the most important features using both classification algorithms, we generated a heatmap of those KO pathways in R using the *pheatmap* library^83^. Further, we calculated the mean relative abundance (±SEM) of KO pathways and used Mann-U t-tests to determine if there were statistically significant differences between estimated postmortem intervals.

To determine whether there was an association between microbial structure and predicted functional profiles, we first performed Spearman rank correlations between the predicted functional richness and community stability based on median weighted UniFrac distances (Fig. S8). Only the eyes (<48 h, >49 h PMI) and rectum (<48 h PMI) had significant correlations (P < 0.05), but all had weak relationships (ρ < 0.5). Then we calculated Spearman rank correlations between OTUs and KO pathways for each anatomic area in R using the *Hmisc* library^84^. Taxa (OTU level) that were significantly positively correlated with predicted function (ρ > 0.5; P < 0.05) were then used to assess the potential functional redundancy of the postmortem microbial communities within each anatomic area.

#### Postmortem Microbiome and Antemortem Health Condition Associations

Binomial logistic regressions were used to model statistical associations of the postmortem microbiome with antemortem health conditions based on phylogenetic diversity (PD). Specifically, we were interested in independently evaluating the postmortem microbial communities of two groups of cases that were the most prevalent categories of cause of death: (1) cases with evidence of heart disease detected during autopsy, and (2) cases with death resulting from violent circumstances (e.g., blunt force trauma, gunshot wound, vehicular accident). Heart disease was based on examination of the heart, and medical history. The pathologic examination includes microscopic examination. It is unsurprising that heart disease was a prevalent cause of death category (36.7%), as it is the leading cause of death in the US^21^. Further, cases resulting from violent deaths are another candidate group for comparisons since it was infrequent that violent cases had a co-occurrence of a chronic health condition (Table S6): 3.7% of violent deaths had evidence of heart disease, and 0% of cases with violent deaths had cancer, diabetes, central nervous system disorders, or thyroid conditions.

Cases within the first 24 h, as opposed to the fist 48 h after death, were selected for this analysis as the model results, based on AIC criteria, for phylogenetic diversity of samples collected less than 24 h since death were better than models produced for samples collected less than 48 h PMI (Table S4). Further, we only tested samples from the mouth as this anatomic location had demonstrated the best temporal discrimination of microbial communities for estimated time of death. Based on these criteria we had data from a total of 83 cases for this analysis. Additionally, in order for the taxon to be included in the log_2_ fold change analysis, the median for a taxon had to be detected at a value of at least 10 in the diseased or control group. All analysis and visualization of regressions and odds ratios were performed in R using the *faraway* and *ggplot2* libraries^85,86^.

#### Power analyses

A *post hoc* power analysis was conducted to retrospectively examine the observed power using mean weighted UniFrac distances in G*Power software^87^. Sample size for each anatomic area was tested (Table S5), assuming that community similarities would be assessed with independent estimated PMI groups (<48 h, >49 h).

## Electronic supplementary material

Supplementary Information

S5 Table
