# Supplementary material for: A large-scale survey of the postmortem human microbiome, and its potential to provide insight into the living health condition
Source: Sci Rep. 2018 Apr 10;8:5724. doi: 10.1038/s41598-018-23989-w (PMC5893548; doi:10.1038/s41598-018-23989-w)
Supplement: Supplementary file 1 — Supplementary Information [file 41598_2018_23989_MOESM1_ESM.docx]

Title: A large-scale survey of the postmortem human microbiome, and its potential to provide insight into the living health condition

**Authors:** Jennifer L. Pechal^1^*, Carl J. Schmidt^2,3^, Heather R. Jordan^4^, M. Eric Benbow^1,5,6^*

**Affiliations:**^1^Michigan State University, Department of Entomology, East Lansing, MI 48824.

^2^Wayne County Medical Examiner’s Office, Detroit, MI 48207.

^3^University of Michigan, Department of Pathology, Ann Arbor, MI 48109.

^4^Mississippi State University, Department of Biological Sciences, Mississippi State, MS 39762.

^5^Michigan State University, Department of Osteopathic Medical Specialties, East Lansing, MI 48824.

^6^Michigan State University, Ecology, Evolutionary Biology, and Behavior Program, East Lansing, MI 48824.

* Correspondence to Jennifer L. Pechal (pechalje@msu.edu) and M. Eric Benbow (benbow@msu.edu)

**Author Contributions:** J.L.P. and M.E.B. designed the study, and together with C.J.S. and H.R.J., framed the research questions. C.J.S. collected the data. J.L.P. analyzed the data. All authors discussed results. J.L.P. and M.E.B. wrote the manuscript with contributions and inputs from all authors.

**Competing financial interests**

The authors declare no competing financial interests.

**Supplementary Information**

**
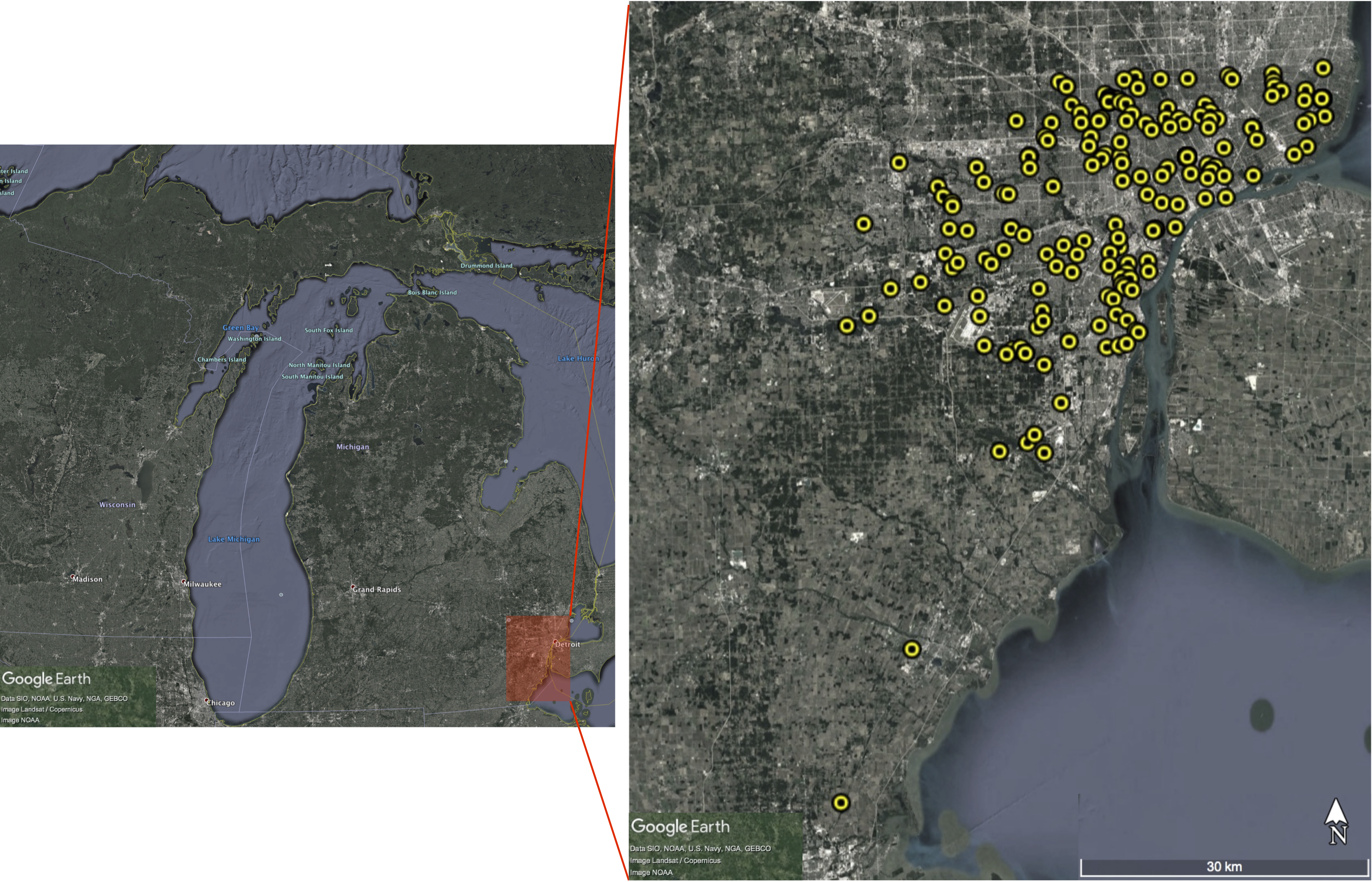
**

**S1 Fig.** **Geographic distribution of cases.** For all 188 cases, each death event location was identified using Google Earth Pro v. 7.1.8.3036 (2017 image copyright NOAA). In certain instances, there are overlapping data points as the deaths from multiple cases occurred at the same location (*e.g.*, hospital or vehicular accident).


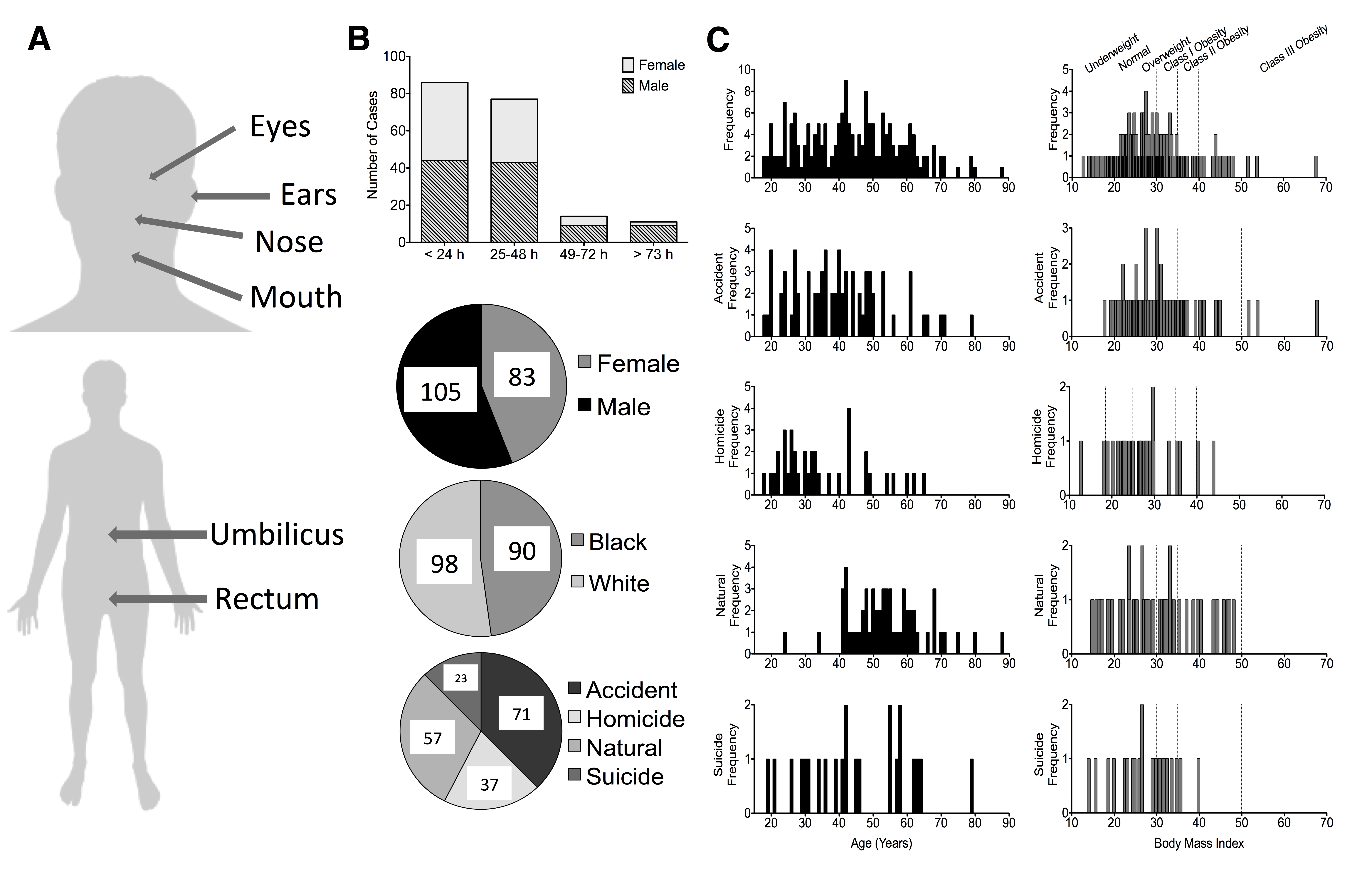


**S2 Fig.** **Summary of anatomic sampling locations and case demographics.** Please see S1-S2 Tables for complete summary of case numbers for each metadata category described in this figure. (**A**) Samples were collected during routine autopsy procedures at the Wayne County Medical Examiner’s Office (Detroit, MI, USA). Microbial DNA was collected from each case at six individual anatomic locations: the eyes, external auditory canal, nose, mouth, umbilicus and rectum. The human silhouette image was modified from a photo created by JE Theriot, available at https://www.flickr.com/photos/jetheriot/7940994640/ and attributed to Creative Commons Attribution 2.0 Generic (CC BY 2.0, https://creativecommons.org/licenses/by/2.0/). (**B**) Estimated postmortem intervals were determined using forensic pathology metrics. There were a nearly balanced proportion of cases between sex and ethnicity, with manners of death mainly resulting from accidental and natural cases. (**c**) Frequency distributions of the number of cases based on age (years) and body mass index (BMI; kg/m^2^) stratified by manner of death (accident, homicide, natural or suicide). BMI for each decedent was classified into obesity classifications as established by the World Health Organization.

**
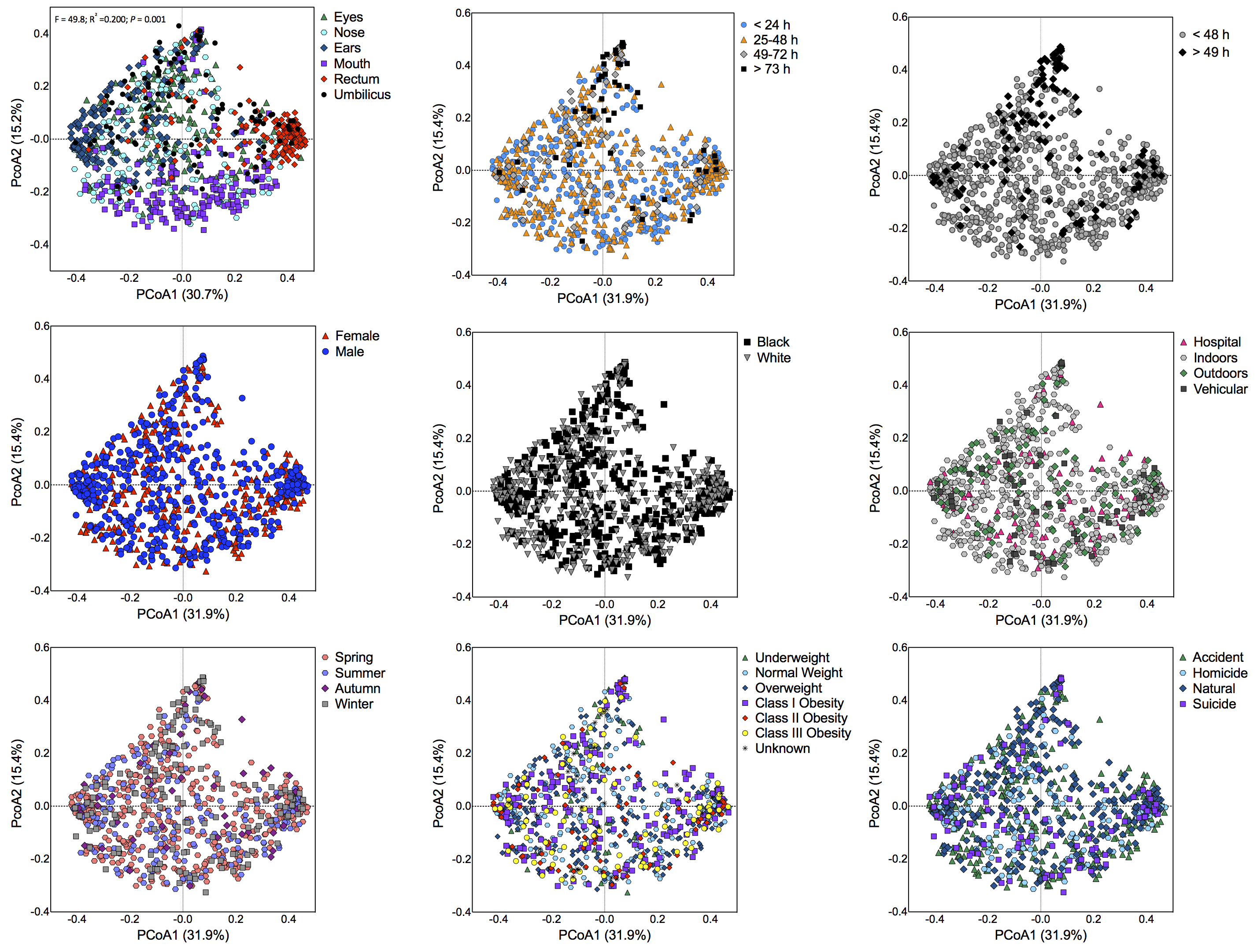
**

**S3 Fig.** **Structuring of the postmortem microbial communities**. Principal coordinates analysis (PCoA) of weighted UniFrac distances for all anatomic regions at the OTU level. Pairwise comparisons from PERMANOVA results indicated anatomic areas were statistically significant (P < 0.001) with p-value adjusted for FDR, thus suggesting differences in the postmortem microbial communities based on body habitat. A PCoA of weighted UniFrac distances was then performed for each metadata category (*i.e.,* sex, ethnicity, death event location, season, weight class, and manner of death) of the cases after the umbilicus samples had been removed from the dataset. PERMANOVA results indicated that there were no strong statistically significant differences (P < 0.001) for any of these metadata covariates. Each axis is annotated with the percentage of total variation explained.


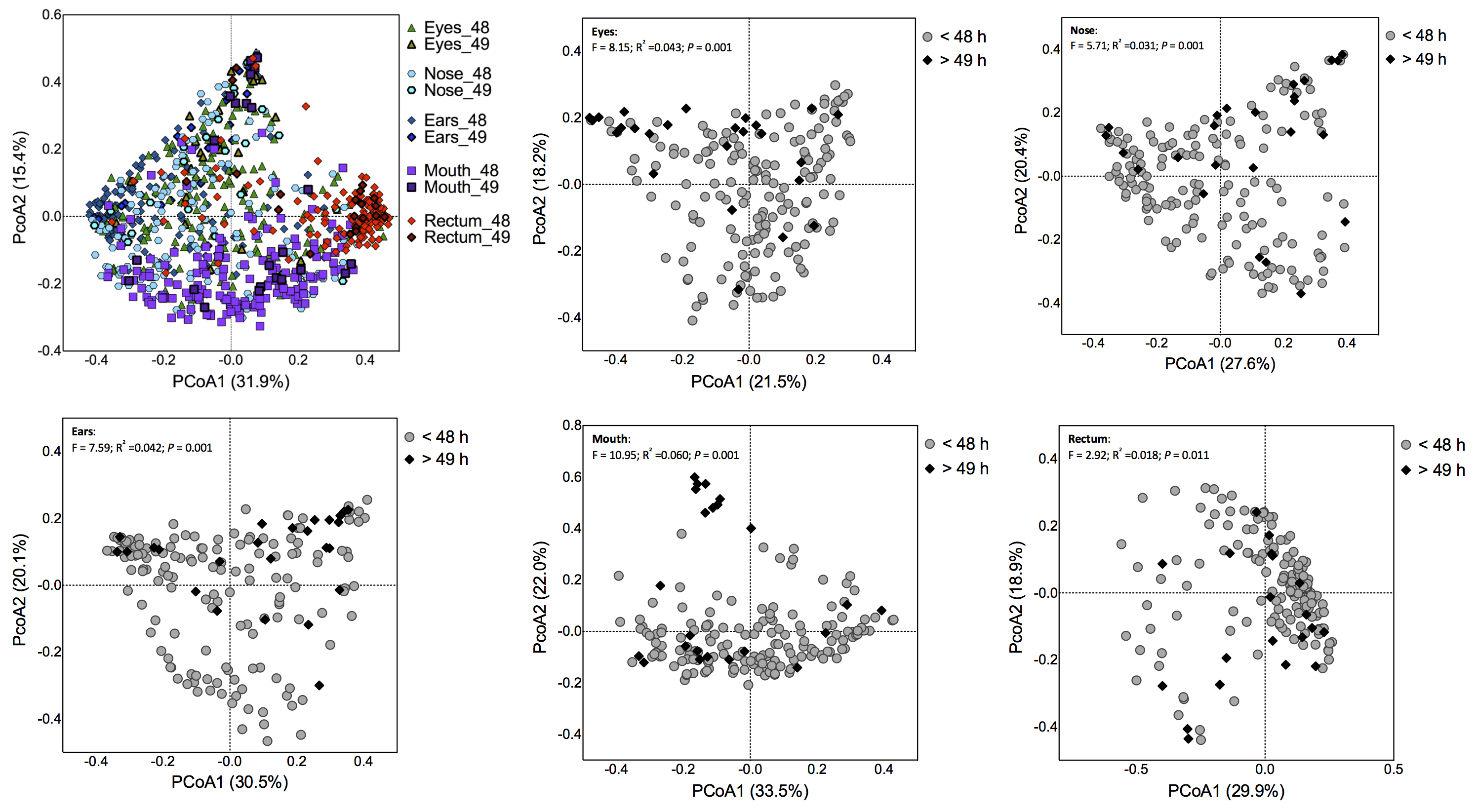


**S4 Fig. Temporal differences of postmortem microbial communities.** Principal coordinates analysis (PCoA) of weighted UniFrac distances to test for differences in the postmortem interval estimates. Pairwise PERMANOVA differences indicated significant differences (P < 0.05) with p-value adjusted for FDR at estimated postmortem interval (PMI) intervals. Initially, the postmortem microbial communities among anatomic areas and time since host death (PMI: < 48 h, > 49 h) were tested for differences in community composition according to weighted UniFrac distances. There were significant differences in both anatomic location and estimated PMI and a significant interaction effect (P < 0.01). Then differences were tested within anatomic location at the broad PMI scale (< 48 h, > 49 h) and we determined all pairwise differences were significantly different (P < 0.001). Each axis is annotated with the percentage of total variation explained.

**
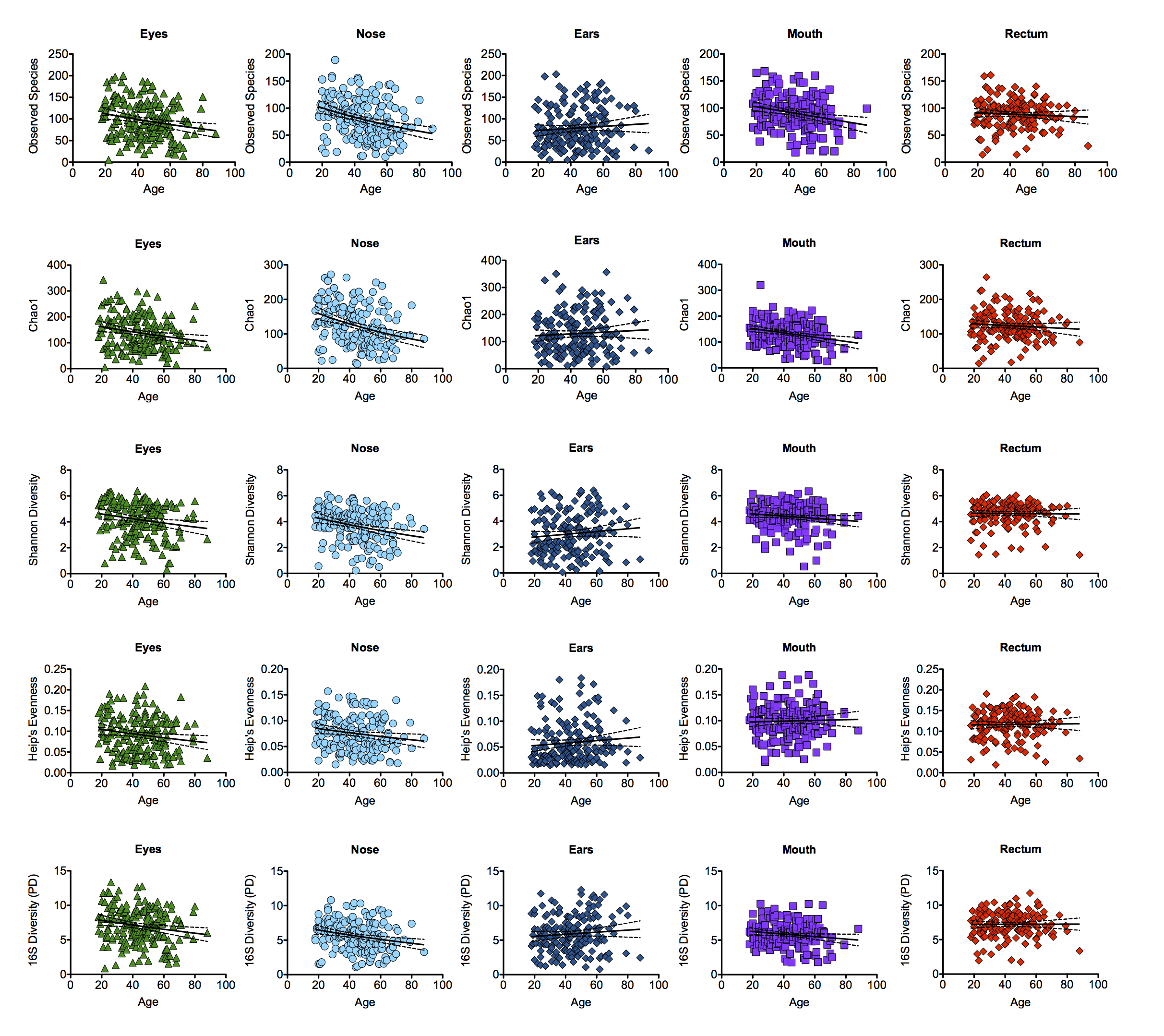
**

**S5 Fig. Trends in microbial community diversity metrics relative to age.** The age of the decedent (years) was regressed against each alpha-diversity metric calculated in QIIME within anatomic area: observed species, Chao1, Shannon-Wiener diversity, Heip’s evenness, and 16S rRNA (Faith’s phylogenetic) diversity. The solid line is the regression and dashed lines are 95% confidence intervals.

**
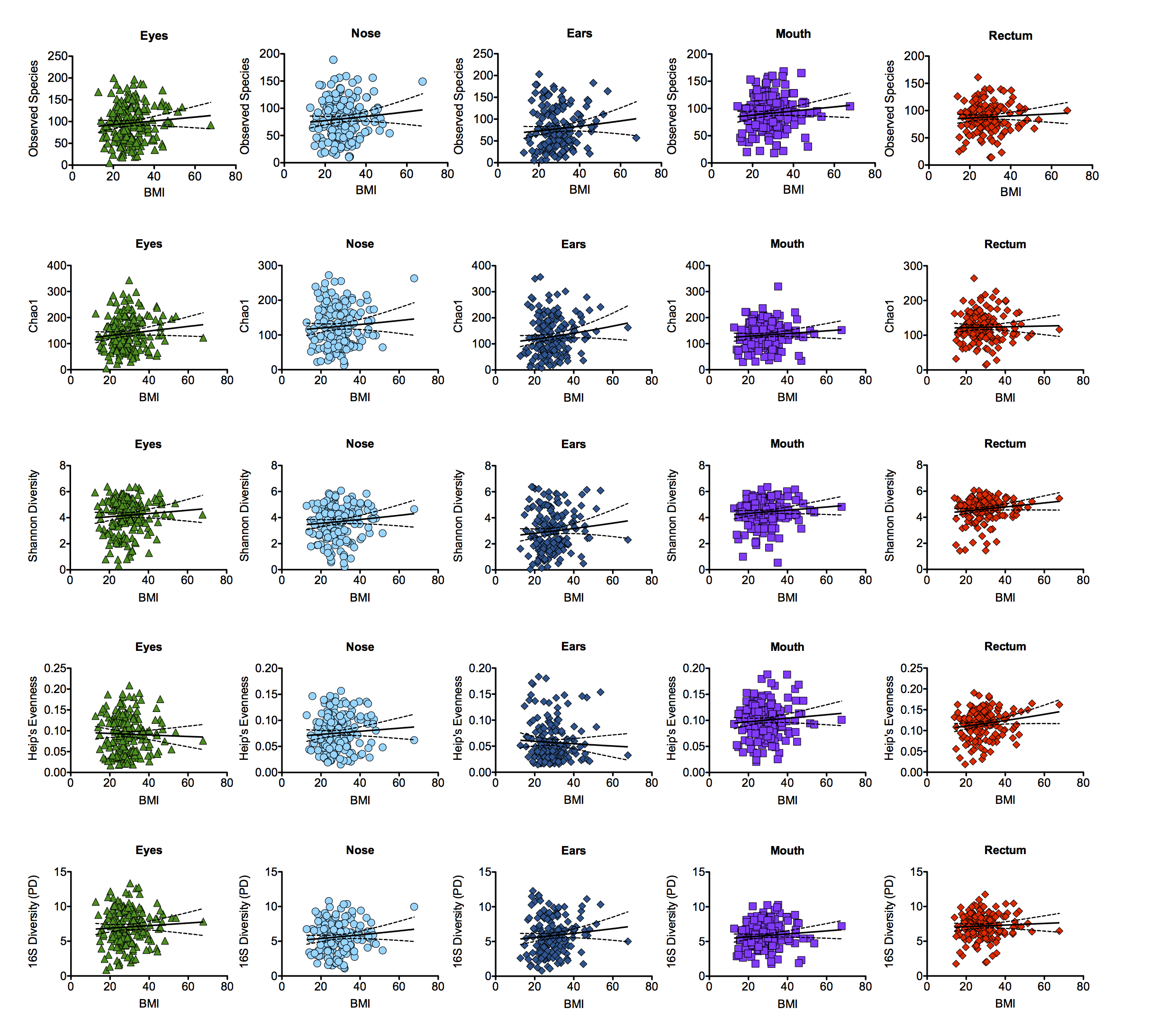
S6 Fig. Trends in microbial community diversity metrics relative to weight status**. The body mass index (BMI: kg/m^2^) was regressed against each alpha-diversity metric calculated in QIIME within anatomic area: observed species, Chao1, Shannon-Wiener diversity, Heip’s evenness, and 16S rRNA (Faith’s phylogenetic) diversity. The solid line is the regression and dashed lines are 95% confidence intervals

**
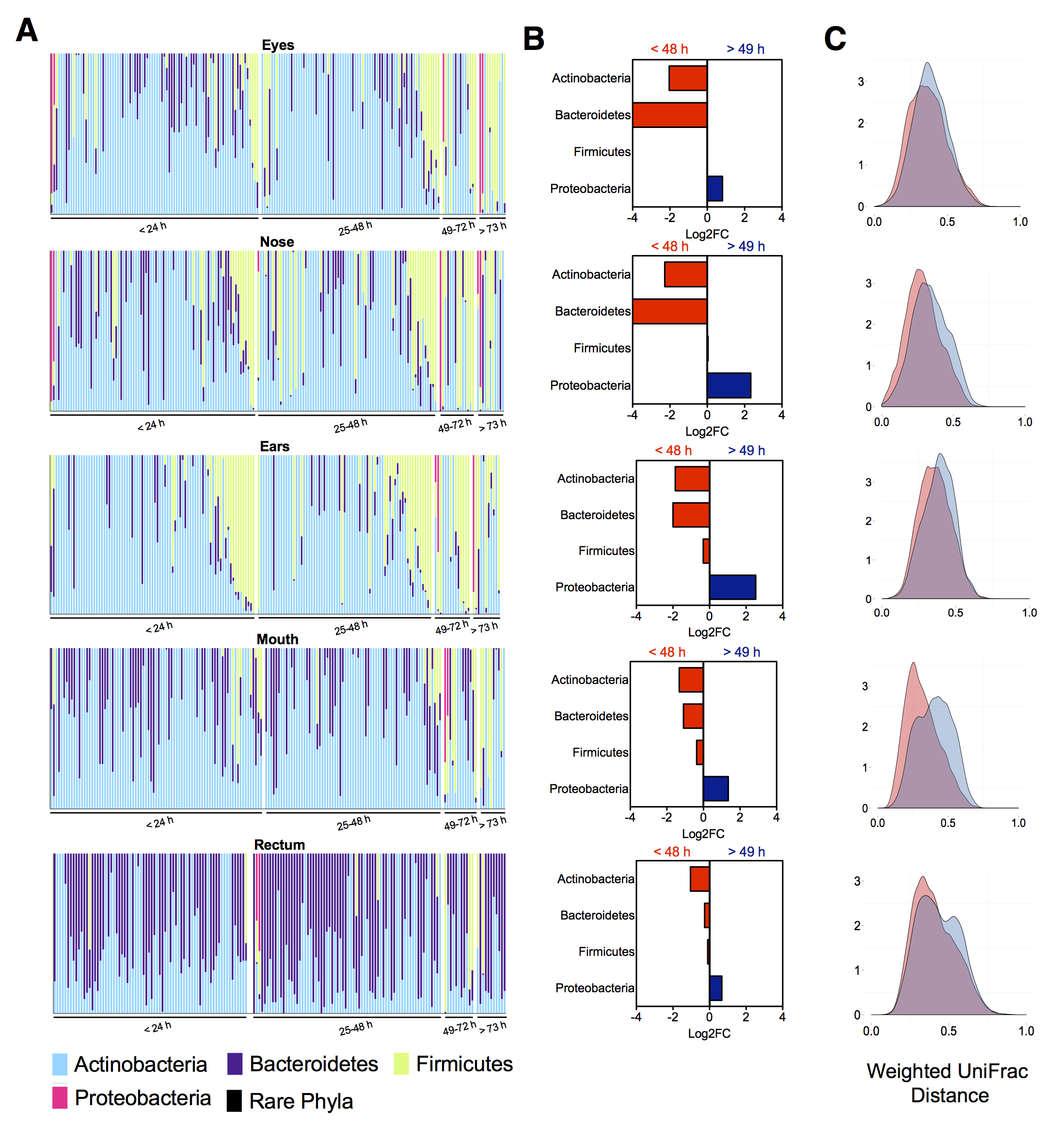
**

**S7 Fig. Taxonomic shifts in the postmortem microbial communities.** (**A**) Relative abundance (% total of rarified sequences) for each sample characterized by 16S rRNA sequencing at the phyletic OTU level. The top four most abundant phyla were visualized with all of the remaining phyla cumulatively summed to create the fifth group of “Rare Phyla”. Each column represents an individual case within each anatomic location with an increase in estimated postmortem interval estimate from left to right. (**C**) Density plots comparing the distribution of weighted UniFrac distances between postmortem interval estimates among anatomic sampling locations assessed the community stability of postmortem microbial communities. The postmortem intervals are colored based on estimated time after host death: red = < 48 h and blue = > 49 h.

**
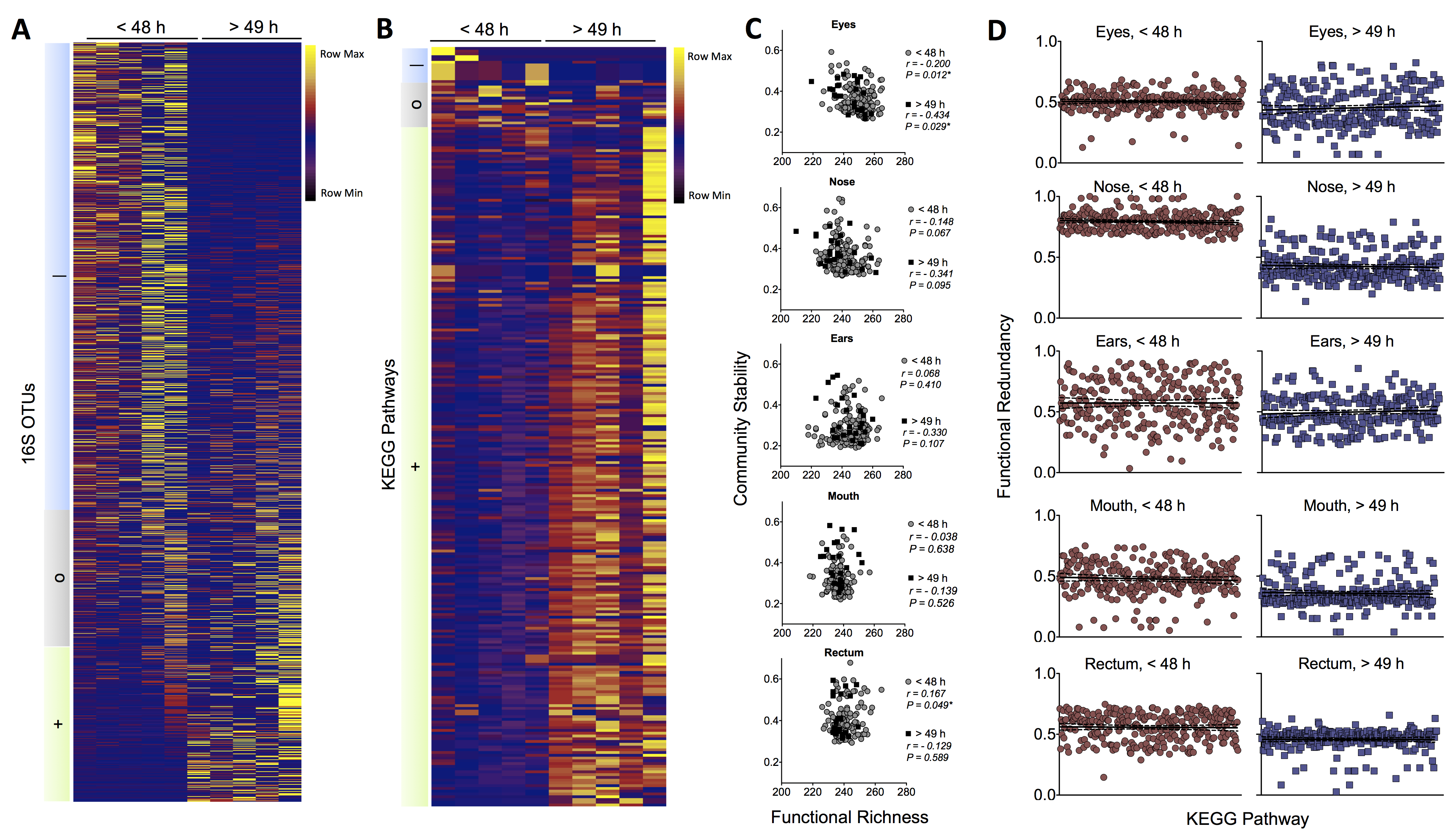
**

**S8 Fig.** **Temporal heterogeneity and relationship between community structure and predicted functional profiles**. Heatmaps of the (**A**) mean relative abundance of OTUs and (**B**) KEGG pathways as decomposition progressed. Each column represents an individual body location from left to right (eyes, nose, ears, mouth, and rectum) within each estimated postmortem interval (less than or greater than 2 days since host death). (**C**) Relationships between postmortem microbial community stability and predicted function. Nonparametric correlations (Spearman’s rank) were performed between the predicted functional richness and community stability based on median weighted UniFrac distances for each anatomic area for groups with estimated intervals of less than or greater than two days after host death. Significant correlations are indicated with an asterisk. (**D**) We assessed functional redundancy by identifying the relative abundance of OTUs positively correlated (Spearman’s rank) with predicted function (KO pathways). The microbiota of the rectum demonstrated the most stable functional link to community composition across postmortem intervals, while the nose and mouth had the most divergent redundancy with a significant decrease (Mann-U t-test, P <0.001) as decomposition progressed.

**S1 Table.** **Summary of case metadata stratified by age**.

|  | **18-30 years** | | **31-45 years** | | **46-55 years** | | **55+ years** |
| --- | --- | --- | --- | --- | --- | --- | --- |
| *n = 188 total (100 %)* | | *41 (22 %)* | | *61 (32 %)* | | *44 (23 %)* | *42 (22 %)* |
| *Sex* | |  | |  | |  |  |
| Female [*n = 83 (44%)]* | | 17 *(9 %)* | | 29 *(15 %)* | | 20 *(11 %)* | 17 *(9 %)* |
| Male [*n = 105 (56%)]* | | 24 *(13 %)* | | 32 *(17 %)* | | 24 *(13 %)* | 25 *(13 %)* |
| *Ethnicity* | |  | |  | |  |  |
| Black [*n = 90 (48%)]* | | 25 *(13 %)* | | 25 *(13 %)* | | 20 *(11 %)* | 20 *(11 %)* |
| White [*n = 98 (52%)]* | | 16 *(9 %)* | | 36 *(19%)* | | 24 *(13%)* | 22 *(12 %)* |
| *Event Location* | |  | |  | |  |  |
| Indoors [*n = 153 (81%)]* | | 24 *(13 %)* | | 57 *(30 %)* | | 39 *(21 %)* | 33 *(18 %)* |
| Outdoors [*n = 24 (13%)]* | | 11 *(6 %)* | | 2 *(1 %)* | | 4 *(2 %)* | 7 *(4 %)* |
| Vehicular [*n = 11 (6%)]* | | 6 *(3 %)* | | 2 *(1 %)* | | 1 *(1 %)* | 2 *(1 %)* |
| *Postmortem Interval Estimate (PMI)* | |  | |  | |  |  |
| < 24 h [*n = 86 (46%)]* | | 22 *(12 %)* | | 27 *(14 %)* | | 20 *(11 %)* | 17 *(9 %)* |
| 25-48 h [*n = 77 (41%)]* | | 17 *(9 %)* | | 27 *(14 %)* | | 18 *(10 %)* | 15 *(8 %)* |
| 49-72 h [*n = 14 (7%)]* | | 1 *(1 %)* | | 6 *(3 %)* | | 4 *(2 %)* | 3 *(2 %)* |
| > 73+ [*n = 11 (6%)]* | | 1 *(1 %)* | | 1 *(1 %)* | | 2 *(1 %)* | 7 *(4 %)* |
| *Weight Status*  *[Body Mass Index (kg/m^2^)]* | |  | |  | |  |  |
| Underweight (< 18.5)  [*n = 12 (6%)]* | | 1 *(1 %)* | | 2 *(1 %)* | | 5 *(3 %)* | 4 *(2 %)* |
| Normal Weight (18.5 – 24.9)  [*n = 48 (26%)]* | | 14 *(7 %)* | | 12 *(6 %)* | | 7 *(4 %)* | 15 *(8 %)* |
| Overweight (25.0 – 29.9)  [*n = 51 (27%)]* | | 12 *(6 %)* | | 16 *(9 %)* | | 14 *(7 %)* | 9 *(5 %)* |
| Class I Obesity (30.0 – 34.9)  [*n = 37 (20%)]* | | 5 *(3 %)* | | 13 *(7 %)* | | 13 *(7%)* | 6 *(3 %)* |
| Class II Obesity (35.0 – 39.9)  [*n = 16 (9%)]* | | 4 *(2 %)* | | 7 *(4 %)* | | 4 *(2 %)* | 1 *(1 %)* |
| Class III Obesity (40.0 – 49.9)  [*n = 18 (10%)]* | | 2 *(1 %)* | | 11 *(6 %)* | | 1 *(1 %)* | 4 *(2 %)* |
| Unknown  [*n = 6 (3%)]* | | 3 *(2 %)* | | -- | | - | 3 *(2 %)* |
| *Season* | |  | |  | |  |  |
| Spring [*n = 104 (55%)]* | | 22 *(12 %)* | | 33 *(18 %)* | | 26 *(14 %)* | 23 *(12 %)* |
| Summer [*n = 44 (23%)]* | | 10 *(5 %)* | | 13 *(7 %)* | | 8 *(4 %)* | 11 *(6 %)* |
| Autumn [*n = 10 (5%)]* | | 3 *(2 %)* | | 4 *(2 %)* | | 2 *(1 %)* | 1 *(1 %)* |
| Winter [*n = 32 (17%)]* | | 6 *(3 %)* | | 11 *(6 %)* | | 8 *(4 %)* | 7 *(4 %)* |
| *Manner of Death* | |  | |  | |  |  |
| Accident [*n = 71 (38%)]* | | 18 *(10 %)* | | 30 *(16 %)* | | 14 *(7 %)* | 9 *(5 %)* |
| Homicide [*n = 37 (20%)]* | | 17 *(9 %)* | | 12 *(6 %)* | | 4 *(2 %)* | 4 *(2 %)* |
| Natural [*n = 57 (30%)]* | | 1 *(1 %)* | | 11 *(6 %)* | | 23 *(12 %)* | 22 *(12 %)* |
| Suicide [*n = 23 (12%)]* | | 5 *(3 %)* | | 8 *(4 %)* | | 3 *(2 %)* | 7 *(4 %)* |

**S2 Table. Detailed summary of cases by postmortem death interval.**

| *n = 188 total (100 %)* | < 24 h  *86 (46%)* | 25-48 h  *77 (41%)* | 49-72 h  *14 (7%)* | > 73+  *11 (6%)* |
| --- | --- | --- | --- | --- |
| *Sex* |  |  |  |  |
| Female [*n = 83 (44%)]* | 42 *(22 %)* | 34 *(18 %)* | 5 *(3 %)* | 2 *(1 %)* |
| Male [*n = 105 (56%)]* | 44 *(23%)* | 43 *(23 %)* | 9 *(5 %)* | 9 *(5 %)* |
| *Ethnicity* |  |  |  |  |
| Black [*n = 90 (48%)]* | 42 *(22 %)* | 35 *(19 %)* | 6 *(3 %)* | 7 *(4 %)* |
| White [*n = 98 (52%)]* | 44 *(23%)* | 42 *(22 %)* | 8 *(4 %)* | 4 *(2 %)* |
| *Event Location* |  |  |  |  |
| Indoors [*n = 153 (81%)]* | 63 *(33 %)* | 68 *(36 %)* | 13 *(7 %)* | 10 *(5 %)* |
| Outdoors [*n = 24 (13%)]* | 16 *(9 %)* | 7 *(4 %)* | -- | 1 *(1 %)* |
| Vehicular [*n = 11 (6%)]* | 8 *(4 %)* | 2 *(1 %)* | 1 *(1 %)* | -- |
| *Season* |  |  |  |  |
| Spring [*n = 104 (55%)]* | 42 *(22 %)* | 45 *(24 %)* | 10 *(5 %)* | 7 *(4 %)* |
| Summer [*n = 44 (23%)]* | 13 *(7 %)* | 24 *(13 %)* | 2 *(1 %)* | 3 *(2 %)* |
| Autumn [*n = 10 (5%)]* | 7 *(4 %)* | 2 *(1 %)* | -- | 1 *(1 %)* |
| Winter [*n = 32 (17%)]* | 24 *(13 %)* | 6 *(3 %)* | 2 *(1 %)* | -- |
| *Manner of Death* |  |  |  |  |
| Accident [*n = 71 (38%)]* | 32 *(17 %)* | 30 *(16 %)* | 6 *(3 %)* | 3 *(2 %)* |
| Homicide [*n = 37 (20%)]* | 22 *(12 %)* | 13 *(7 %)* | 1 *(1 %)* | 1 *(1 %)* |
| Natural [*n = 57 (30%)]* | 20 *(11 %)* | 25 *(13 %)* | 5 *(3 %)* | 7 *(4 %)* |
| Suicide [*n = 23 (12%)]* | 12 *(6 %)* | 9 *(5 %)* | 2 *(1 %)* | -- |

**S3 Table.** Results from permutational multivariate analysis of variance (PERMANOVA) tests of full models on weighted generalized UniFrac distance matrix. The full model includes all metadata collected for each case using a fine scale postmortem interval estimate (< 24 h, 25-48 h, 49-72 h, > 73 h) and broad scale postmortem interval estimate (< 48 h, > 49 h). Significant results (P < 0.05) are indicated by an asterisk.

| **Postmortem Interval Scale** | **Factor** | **d.f** | **SS** | **MS** | **F** | **R^2^** | **P** |
| --- | --- | --- | --- | --- | --- | --- | --- |
| Fine | Fine PMI | 3 | 0.385 | 0.128 | 0.835 | 0.002 | 0.669 |
| (<24 h, 25-48 h, | Anatomic Area | 4 | 35.6 | 8.90 | 57.94 | 0.211 | 0.001 * |
| 49-72 h, >73 h) | Sex | 1 | 0.162 | 0.162 | 1.06 | 0.001 | 0.342 |
|  | Ethnicity | 1 | 0.111 | 0.111 | 0.722 | 0.001 | 0.617 |
|  | Event Location | 3 | 0.428 | 0.143 | 0.928 | 0.003 | 0.539 |
|  | Season | 3 | 0.372 | 0.124 | 0.807 | 0.002 | 0.735 |
|  | Weight Status | 7 | 0.844 | 0.121 | 0.785 | 0.005 | 0.873 |
|  | Manner of Death | 3 | 0.611 | 0.204 | 1.33 | 0.004 | 0.119 |
|  | Residuals | 848 | 130.2 | 0.154 |  | 0.771 |  |
|  | Total | 873 | 168.8 |  |  | 1.00 |  |
| Broad | Broad PMI | 1 | 0.134 | 0.134 | 0.871 | 0.001 | 0.481 |
| (< 48 h, > 49 h) | Anatomic Area | 4 | 35.6 | 8.90 | 57.97 | 0.211 | 0.001 * |
|  | Sex | 1 | 0.162 | 0.162 | 1.06 | 0.001 | 0.354 |
|  | Ethnicity | 1 | 0.105 | 0.105 | 0.681 | 0.001 | 0.688 |
|  | Event Location | 3 | 0.430 | 0.143 | 0.934 | 0.003 | 0.533 |
|  | Season | 3 | 0.344 | 0.115 | 0.748 | 0.002 | 0.761 |
|  | Weight Status | 7 | 0.859 | 0.123 | 0.799 | 0.005 | 0.824 |
|  | Manner of Death | 3 | 0.603 | 0.201 | 1.310 | 0.004 | 0.156 |
|  | Residuals | 850 | 130.5 | 0.154 |  | 0.773 |  |
|  | Total | 873 | 168.7 |  |  | 1.00 |  |

**S4 Table. ANCOM Results.** Summary of differential abundant taxa results using the analysis of community (ANCOM) test for each anatomic location and estimated postmortem intervals (PMI). Estimated PMI ranges are either “fine” (< 24 h, 25-48 h, 49-72 h, > 73 h) or “broad” sale (< 48 h, > 49 h). All listed significant features [operational taxonomic unit (OTU)] rejected the null hypothesis.

| **Anatomic Area** | **Estimated PMI** | ***W* statistic** | **Centered Log-ratio Mean Difference** | **Highest Percentile Abundance Group** | **Significant Feature (OTU)** |
| --- | --- | --- | --- | --- | --- |
| Ears | Fine | 852 | 15.772 | > 73 | k__Bacteria; p__Proteobacteria; c__Gammaproteobacteria; o__Enterobacteriales; f__Enterobacteriaceae; g__; s__ |
|  |  | 814 | 18.606 | > 73 | k__Bacteria; p__Firmicutes; c__Erysipelotrichi; o__Erysipelotrichales; f__Erysipelotrichaceae; g__Erysipelothrix; s__ |
|  |  | 864 | 9.988 | 49-72 | k__Bacteria; p__Firmicutes; c__Clostridia; o__Clostridiales; f__Clostridiaceae; g__Clostridium; s__ |
| Eyes | Broad | 960 | 0.956 | > 49 | k__Bacteria; p__Proteobacteria; c__Gammaproteobacteria; o__Enterobacteriales; f__Enterobacteriaceae; g__Proteus; s__ |
| Eyes | Fine | 988 | 17.247 | > 73 | k__Bacteria; p__Firmicutes; c__Clostridia; o__Clostridiales; f__Clostridiaceae; g__Clostridium; s__ |
|  |  | 1013 | 9.454 | < 24, 25-48 | k__Bacteria; p__Firmicutes; c__Bacilli; o__Lactobacillales; f__Streptococcaceae; g__Streptococcus; s__ |
| Mouth | Broad | 719 | 0.801 | > 49 | k__Bacteria; p__Firmicutes; c__Bacilli; o__Lactobacillales; f__Lactobacillaceae; g__Lactobacillus; s__zeae |
|  |  | 684 | 0.688 | > 49 | k__Bacteria; p__Proteobacteria; c__Gammaproteobacteria; o__Enterobacteriales; f__Enterobacteriaceae; g__Proteus; s__ |
| Mouth | Fine | 734 | 9.259 | < 24, 25-48 | k__Bacteria; p__Proteobacteria; c__Gammaproteobacteria; o__Pasteurellales; f__Pasteurellaceae; g__Haemophilus; s__parainfluenzae |
|  |  | 759 | 14.86 | < 24, 25-48 | k__Bacteria; p__Firmicutes; c__Bacilli; o__Lactobacillales; f__Streptococcaceae; g__Streptococcus; s__ |
| Nose | Fine | 955 | 18.414 | > 73 | k__Bacteria; p__Firmicutes; c__Clostridia; o__Clostridiales; f__Clostridiaceae; g__Clostridium; s__ |
|  |  | 895 | 10.564 | > 73 | k__Bacteria; p__Proteobacteria; c__Gammaproteobacteria; o__Xanthomonadales; f__Xanthomonadaceae; g__Ignatzschineria; s__ |
| Rectum | Fine | 573 | 8.379 | 25-49, 49-72 | k__Bacteria; p__Firmicutes; c__Clostridia; o__Clostridiales; f__Lachnospiraceae; g__; s__ |

**S5 Table. Gradient boosting model performance.** Model performance of stochastic gradient boosting regressions are downloadable in the supplemental excel file.

**S6 Table. Demographic summary of the antemortem health condition related to the cases.**

| **Antemortem Health Condition** | **# of Cases** | **Sex Ratio (Males: Females** | **Ethnicity Ratio (Black: White** | **Median Age (Years Range)** | **Black, Female Median Age**  **[# cases]** | **Black, Male Median Age**  **[# cases]** | **White, Female Median Age**  **[# cases]** | **White, Male Median Age**  **[# cases]** |
| --- | --- | --- | --- | --- | --- | --- | --- | --- |
| Heart Disease | 69 | 31:38 | 33:36 | 53  (23-88) | 47  [15] | 55  [18] | 51  [16] | 53  [20] |
| No Heart Disease | 119 | 52:67 | 57:62 | 38  (18-75) | 43  [24] | 32  [33] | 41  [28] | 38  [34] |
| Violence | 67 | 29:38 | 38:29 | 38  (18-79) | 41  [14] | 28  [24] | 45  [15] | 47  [14] |
| No Violence | 121 | 54:66 | 52:69 | 46  (19-88) | 47  [25] | 53  [27] | 42  [29] | 43  [40] |
| Heart Disease  + Violence | 7 | 3:4 | 2:5 | 57  (33-79) | 39  [1] | 33  [2] | 79  [1] | 57  [3] |
| Heart Disease  + No Violence | 62 | 27:34 | 31:31 | 53  (23-88) | 50  [14] | 55  [17] | 48  [14] | 52  [17] |
| No Heart Disease + Violence | 60 | 26:34 | 36:24 | 36  (18-71) | 42  [13] | 27  [23] | 42  [13] | 46  [11] |
| No Heart Disease + No Violence | 59 | 26:33 | 21:38 | 39  (19-75) | 44  [11] | 43  [10] | 35  [15] | 36  [23] |

**S7 Table.** The results from binomial logistic regression models using phylogenic diversity (PD) of the mouth microbial communities as a predictor of estimated time since host death (< 24 h PMI versus < 48 PMI). Specifically, these analyses were performed in cases with a chronic antemortem health condition (heart disease) or resulting from a violent death. Model performance was determined from Akaike information criterion (AIC) scores. Significant results (P < 0.05) are indicated by an asterisk.

| **Antemortem Health Condition** | **Estimated Time Since Death** | **# Cases (Absent/**  **Present)** | **AIC** | **Coefficient** | **Estimate** | **Std. Error** | **z** | **Pr(>\|z\|)** |
| --- | --- | --- | --- | --- | --- | --- | --- | --- |
| Heart Disease | < 24 h | 83 | 98.989 | Intercept | 1.151 | 0.990 | 1.16 | 0.245 |
|  |  | (59/24) |  | PD | -0.339 | 0.164 | -2.07 | 0.038 * |
| Heart Disease | < 48 h | 152 | 193.93 | Intercept | 0.555 | 0.645 | 0.86 | 0.390 |
|  |  | (101/51) |  | PD | -0.209 | 0.107 | -1.96 | 0.050 * |
| Violence | < 24 h | 83 | 105.24 | Intercept | -3.387 | 1.052 | -3.22 | 0.001* |
|  |  | (41/42) |  | PD | 0.550 | 0.167 | 3.30 | < 0.001* |
| Violence | < 48 h | 152 | 198.38 | Intercept | -2.451 | 0.682 | -3.59 | < 0.001* |
|  |  | (90/62) |  | PD | 0.341 | 0.107 | 3.18 | < 0.001* |

**S8 Table.** A post hoc analysis estimated the observed (two-tailed hypothesis) power of the analysis for an estimated postmortem interval of less than or greater than two days after host death for each anatomic area.

| **Anatomic Area** | **Calculated d  (effect size)** | **alpha** | **Sample size  Group 1 (< 48 PMI)** | **Sample size  Group 2 (> 49 PMI)** | **Noncentrality  parameter** | **Critical T** | **DF** | **Power (1-beta)** |
| --- | --- | --- | --- | --- | --- | --- | --- | --- |
| Ears | 0.62 | 0.05 | 150 | 25 | 2.81 | 1.97 | 165.1 | 0.80 |
| Eyes | 0.41 | 0.05 | 158 | 25 | 1.86 | 1.97 | 172.8 | 0.46 |
| Nose | 0.27 | 0.05 | 154 | 25 | 1.23 | 1.97 | 168.9 | 0.23 |
| Mouth | 1.13 | 0.05 | 152 | 23 | 4.94 | 1.97 | 165.1 | 1.00 |
| Rectum | 0.32 | 0.05 | 142 | 20 | 1.31 | 1.98 | 152.7 | 0.26 |
